# Toward a Consolidated Lignin Biorefinery: Preserving the Lignin Structure through Additive‐Free Protection Strategies

**DOI:** 10.1002/cssc.202000974

**Published:** 2020-06-30

**Authors:** Maria Karlsson, Nicola Giummarella, Pär A. Lindén, Martin Lawoko

**Affiliations:** ^1^ Wallenberg Wood Science Center, Department of Fiber and Polymer, Technology School of Chemistry Royal Institute of Technology, KTH Teknikringen 56–58 100 44 Stockholm Sweden; ^2^ Division of Wood Chemistry and Pulp technology, Department of Fiber and Polymer Technology School of Chemistry Royal Institute of Technology, KTH Teknikringen 56–58 100 44 Stockholm Sweden

**Keywords:** biorefinery, green chemistry, lignin, NMR spectroscopy, static cycles

## Abstract

As part of the continuing efforts in lignin‐first biorefinery concepts, this study concerns a consolidated green processing approach to obtain high yields of hemicelluloses and lignin with a close to native molecular structure, leaving a fiber fraction enriched in crystalline cellulose. This is done by subcritical water extraction of hemicelluloses followed by organosolv lignin extraction. This initial report focuses on a detailed characterization of the lignin component, with the aim of unravelling processing strategies for the preservation of the native linkages while still obtaining good yields and high purity. To this effect, a static cycle process is developed as a physical protection strategy for lignin, and advanced NMR analysis is applied to study structural changes in lignin. Chemical protection mechanisms in the cyclic method are also reported and contrasted with the mechanisms in a reference batch extraction process where the role of homolytic cleavage in subsequent repolymerization reactions is elucidated.

## Graphical abstract

**Powers of consolidation**: A consolidated green processing approach has been developed to obtain high yields of hemicelluloses and lignin with a close to native molecular structure, by subcritical water extraction of hemicelluloses followed by organosolv lignin extraction. A static cycle process is developed as a physical protection strategy for lignin with advanced NMR analysis to monitor any structural changes in the lignin.

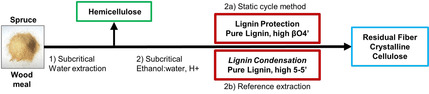

## Introduction

Combating climate change has become a global initiative,^1^ and a strong case can be made for lignocellulosic biomass to replace fossil sources as a raw material.^2^ Lignin accounts for approximately 15–30 % of lignocellulosic biomass and is the most abundant natural source of aromatics.^3^ From a sustainability and bioeconomical viewpoint, replacing fossil‐based aromatics with sustainable solutions is of high interest where lignin could be used as a precursor for biofuel and materials.^4^ However, the fractionation recalcitrance of biomass to obtain biopolymers is an obstacle that still demands careful evaluation.

One of the conventional pulping processes is the organosolv process, which was patented by Kleinert in 1971.^5^ The working principle of the organosolv process is to use an aqueous organic solvent to extract lignin, where low molecular weight aliphatic alcohols are especially used for the extraction. Primary alcohols have shown more selective delignification than secondary and tertiary alcohols.^6^ The lower viscosity of organic solvents makes the solvent dispersion in the wood faster. The process can also be performed with catalysts as well as additives with different catalysts having been shown to enhance the extraction of lignin.^7^

One of the most promising organosolv processes is the Alcell process, which is based on the biorefinery concept that pulp, lignin, furfurals, acetic acid, and hemicelluloses could all be of value.^8^ The Alcell process was developed for hardwoods by the Canadian pulp and paper industry.^9^ However, the principle of using aqueous ethanol with a catalyst was already investigated in the early 1970s in small‐scale pilot plants.^10^ The principle of the process is described in various patents: Generally, the extraction uses a binary ethanol–water solvent system in the range of 20–80 wt % of alcohol at temperatures of 160–220 °C with no catalyst added.^11^ Ethanol could be recovered by means of distillation, owingto its low boiling point, making it particularly practical from a recovery viewpoint compared to other organic solvents. High quality pulp has been generated from the process.^9^, ^12^

With no catalyst added, the extraction solvent has a pH of around 4, owing to the generation of acetic acid from deacetylation of hemicelluloses. For more efficient lignin extraction, a small amount of mineral acid is commonly added.^13^ A mild organosolv extraction of lignin subsequent to hydrothermal pretreatment was recently reported.^14^ Even more recently, polyhydroxy alcohols, such as butanediol^15^ and ethylene glycol in combination with dimethyl carbonate,^16^ have been used in the reactive dissolution of biorefinery lignins.

The structure of organosolv‐extracted lignin differs from that obtained by other processes. Under acidic conditions and at high temperatures, carbocations formed in the aliphatic side chains are prone to react with electron‐rich aromatic carbons in an electrophilic attack. This reaction contributes to the formation of stable C−C bonds in condensation reactions.^12^ A common depolymerization reaction in organosolv processes is acidolysis and the subsequent formation of Hibbert's ketone.^17^, ^18^

More specifically, occurrence of undesirable condensation reactions resulting from carbocations at alpha carbons has been reported. For these reactions, protection strategies have been proposed. One protection strategy is to use chemical protection, where a protecting group deactivates, reversibly or irreversibly, a reactive functional group.^19^ One example of this strategy is the addition of formaldehyde in the pretreatment of the biomass. Here, upon acetalization, lignin condensation and the formation of stable C−C bonds are prevented by blocking reactive positions prone to lignin condensation. The resulting derivative can be reversibly deactivated to achieve deprotection. Under acidic conditions, the formaldehyde additive can also react with the electron‐rich position *para* to the methoxy group in the aromatic ring of guaiacyl lignin.^20^

Another way to increase the efficiency of the extraction and minimize condensation reactions is to use a physical protection strategy. Flow‐through extraction, a method of continuous extraction, is especially efficient for matrices such as biomass, where components that are extracted in an early step are prone to undergo further reactions in the extract. The principle of the setup is simple; an extraction cell is connected to a pump system that continuously provides new solvent into the cell. The extraction time is short and the sample is thereafter cooled down in a condenser.^19^ The principle follows classical continuous extraction concepts where the extraction liquid is less saturated, which is beneficial for efficient extraction and also limits further reactions in the dissolved fractions. It has previously been reported that β‐O‐4′ units are preserved to a higher degree when using the flow‐through principle, making the lignin produced by this method more suitable for monomer production in a lignin biorefinery.^21^, ^22^, ^23^

Consolidated lignin biorefineries, where value can be derived from all streams, are of increasing interest. With the goal of furthering fundamental understanding in this field, we designed and investigated a two‐step sustainable solvent extraction approach for consolidating lignin biorefining with hemicellulose and fiber production. Softwood, which represents the main technical wood species in Scandinavia, was chosen for this initial study. The concept was to further develop a mild green consolidated extraction process, where subcritical water extraction of hemicelluloses is followed by a lignin extraction based on the solvent system used in the Alcell process,^11^ in this case ethanol–water (70:30 *v*/*v*). Small amounts of sulfuric acid were added to address lignin purity concerns and extraction temperatures were kept lower (160 °C) than those in the Alcell process to minimize lignin modification. The concept was developed according to a group of pre‐set criteria, resulting in a sustainable approach for the extraction of potentially high‐value “native”‐like lignin manifested by a high degree of β‐O‐4′ interunit linkage, a lower degree of condensation, high purity, and high yield.

## Results and Discussion

**Figure 1:**
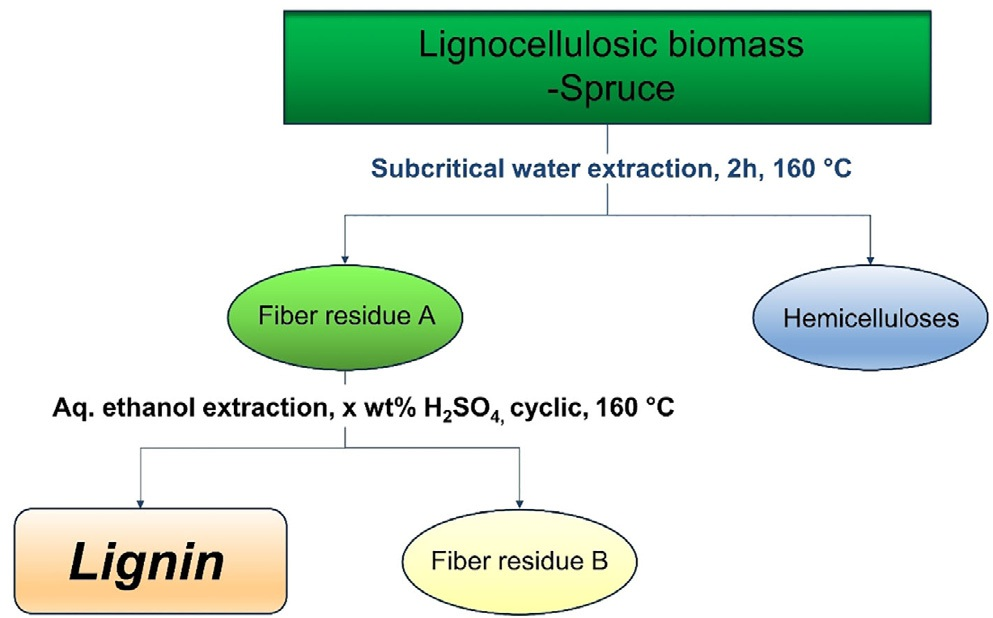
Principle of the consolidated biorefinery concept.

Given the need for better material usage and circularity, consolidated biorefinery concepts are attractive. We have explored a two‐step three‐component strategy, with the initial aim of gaining fundamental understanding of such processes. Solvents were selected in consideration of the principles of green chemistry and circularity. Thus, water and ethanol were chosen. The use of both subcritical water and ethanol in biomass extraction have been previously studied. However, a combination of the two in a sequential operation to fulfil integrated biorefinery needs has to our knowledge not been previously investigated. In this study, we have investigated the potential of this system, with the primary focus of developing the fundamental understanding of lignin reactivity and its control. A scheme representation of the extraction approach is shown in Figure 1.

Subcritical liquids, that is, liquids where the temperature or pressure is slightly under the critical value, have the unique property of simultaneous low viscosity and high diffusivity.^24^ These properties enhance mass transfer and have advantages in addressing biomass recalcitrance to fractionation.

### Hydrothermal extract (hemicellulose) and final fiber residue (fiber B)

**Figure 2:**
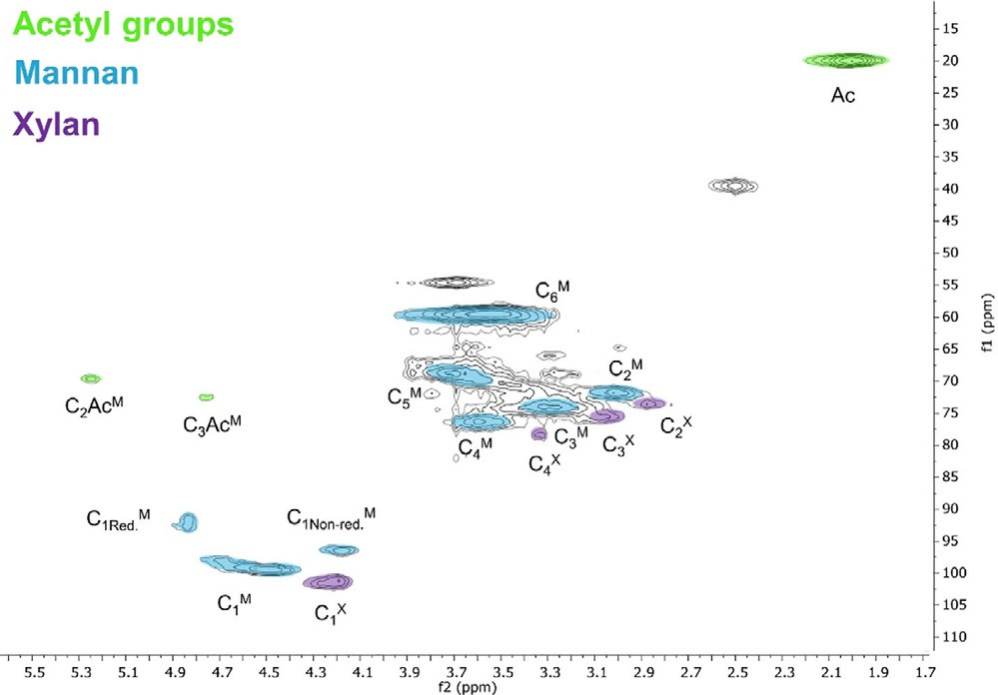
HSQC NMR spectra of the hydrothermal extract. f1: ^13^C, f2: ^1^H. Subscripts indicate the carbon number in accordance with saccharide nomenclature; M=mannan; X=xylan; Ac represents *O*‐acetylated carbons; Red=reducing end; Non‐red=nonreducing end.

In the first step, subcritical water extraction was implemented. Extraction conditions were based on findings from previous work in our research group.^25^ These findings also showed that the extracted hemicelluloses were partially hydrolyzed but still contained glycosidic bonds and acetyl moieties, which are indicative of a mild extraction. Furthermore, when the extraction temperature was set to 160 °C, the formation of pseudo‐lignin from hemicellulose degradation products was negligible.^26^ The molecular weight distribution was studied by size‐exclusion chromatography (SEC; see the Supporting Information, Figure S31). Two populations were observed with respect to molar mass. In the higher molar mass fraction, which accounted for 61 % of the chromatogram area, *M*_n_ and *M*_w_ were 1050 and 3050, respectively, and the dispersity index (*Đ*) was 2.9. The approximate degree of polymerization (DP_n_) as determined by using the anhydromannose unit (162 g mol^−1^) as a repeating unit, was about 6–7. The HSQC spectra of the hemicelluloses extracted in the present study (Figure 2) display native structures indicative of a mild extraction in the form of partially *O*‐acetylated C2 and C3 hydroxy groups.

Taken together, the SEC and HSQC data suggest that the native hemicelluloses are partially hydrolyzed at the glycosidic bond but that the resulting oligomers have preserved their native structures. Such native hemicelluloses have potential in applications such as emulsions^27^ and could also be subsequently fermented to ethanol.^28^ In the latter case, the bioethanol produced could potentially be used in the subsequent organosolv extraction step described herein, thereby contributing to the circularity aspects of the process. The subsequent organosolv extraction is discussed in detail in the next section.

The final fiber residue (fiber B, Figure 1) was also analyzed by X‐ray diffraction (XRD) and, as expected, the crystallinity of cellulose was retained after both extraction steps (Figure S1). This could be of interest for composite applications.^29^ The compositions of the hot water extract and the final fiber were also elucidated by sugar and lignin analyses. Mass balances are discussed in a later section.

### Organosolv (aqueous ethanol) extraction method

**Figure 3:**
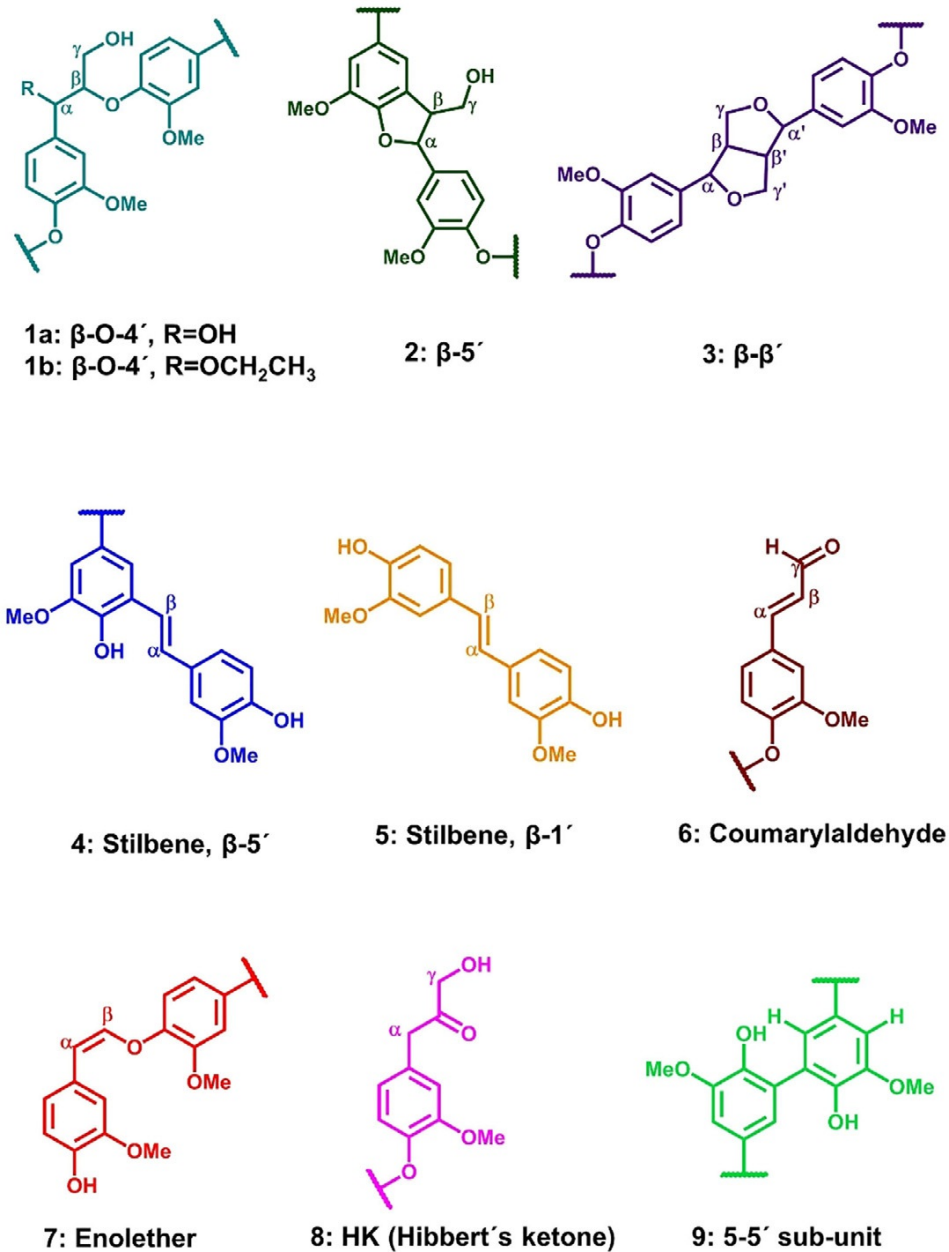
Lignin interunit linkages and substructures from the extraction procedure.

In this work, we will mainly focus on the lignin fraction with the emphasis on achieving the preset criteria mentioned earlier, that is, high yield and purity combined with a predominance of native interunit linkages (Figure 3).

**Figure 4:**
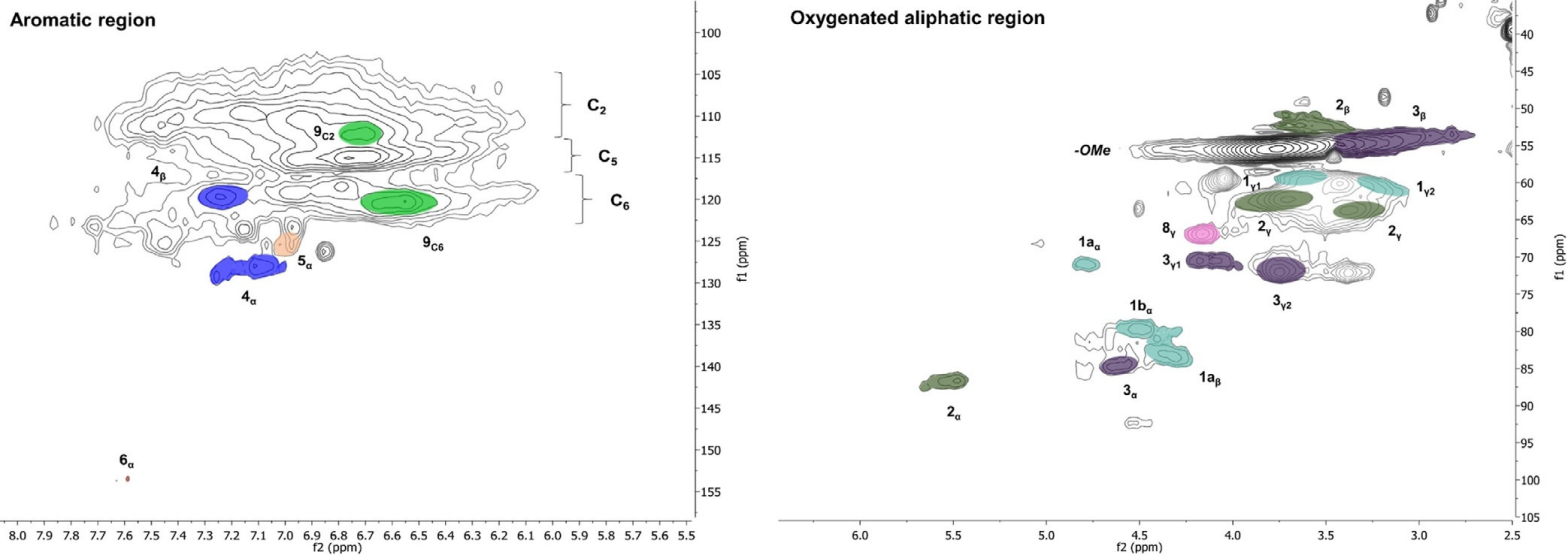
HSQC spectra for reference organosolv extractions. Reaction conditions: 2 h, 160 °C, 1.5 wt % sulfuric acid extraction. f1: ^13^C, f2: ^1^H. Figures are colored and labeled according to the structures depicted in Figure 3.

Our starting point was two reference organosolv extractions with extraction times of 2 h (Figure 4) and 3 h at 160 °C with 1.5 wt % sulfuric acid (for full DEPT‐edited HSQC spectra, see Figures S18 and S19). These conditions reflect common time scales and temperatures for organosolv extractions. The difference here is that most of the hemicelluloses have already been extracted. This may improve the porosity of the material, resulting in faster kinetics and improved selectivity for the lignin extraction. The contents of β‐aryl ether linkages (β‐O‐4′) and other common native linkages were analyzed by HSQC, as an indicator of the mildness of the extraction.

The two reference samples showed, as is typical for most organosolv processes, low contents of β‐O‐4′ bonds (at 7 % for the 2 h sample and 4 % for the 3 h sample; Table 1). In contrast, the β‐O‐4′ content of native spruce lignin has been reported to be in the order of 35–60 %.^30^ Nevertheless, the β‐O‐4′ bonds appeared to be both hydroxylated and etherified at Cα, in agreement with reported results.^31^ Such etherification reactions occur through addition reactions of ethanol to electrophilic benzylic cations and have been postulated to improve the solubility of lignin in ethanol. This is further discussed later in connection to mechanisms. Under the 2 h and 3 h extraction conditions, 64 % and 100 % of the β‐O‐4′ structures were etherified, respectively.

**Table 1:** Quantification of interunit linkages, substructures and total extraction yield (Figure 3). All interunit linkages and substructures are semi‐quantified per 100 Ar units (for diagnostic chemical shifts, see Table S1). For the 0.5 % and 1.5 % acid series, values are presented as mean ±SD where *n*=9 and *n*=5^[a]^ for the 0.5 % series. The number of *n* reflects the number of collected fractions in the trend series investigation. For the 1.5 % series, *n*=5. The high SD for the β‐1′ stilbene structures can be attributed to a downward trend and for the β‐5′ stilbene structures an upward trend from the first to the last fraction. The HSQC spectra for the 1.5 % and 0.5 % acid series are included in the Supporting Information (Figures S3–S7 and Figures S8–S16, respectively).

Fraction	β‐O‐4′	β‐5′	β‐β′	Stilbene, β‐1′	Stilbene, β‐5′	Enol ether	Coumaryl aldehyde	HK^[b]^	Lignin yield [mass %]
2 h	7	6	1.5	0.84	6.11	0.85	0.41	1	56.3
3 h	4	5	1.1	n.d.^[c]^	4.33	1.9	n.d.	0.26	69.4
Cyclic, 1.5 % acid	34±2.9	9.3±1.3	3.1±0.40	2.3±1.5	3.9±0.89	1.9±0.45	1.9±0.21	1.4±0.20	66.9
Cyclic, 0.5 % acid	34±3.1	11±1.1	3.1±0.26	2.3±1.3	2.5±0.69	1.7±0.18	1.7±0.27	1.1±0.23	41.4
Cyclic, 0.5 % acid^[a]^	35±3.7	11±0.94	3.2±0.31	3.1±1.3	2.1±0.64	1.8±0.14	1.9±0.18	1.1±0.32	
Integrated, cyclic, 1.5 % acid	30	12	2.4	4.4	3.9	0.87	2.0	1.3	53.3
MWL, wood	33	12	3.3	n.d.	n.d.	n.d.	n.d.	n.d.	
MWL, after HT extraction	32	14	4.4	n.d.	n.d.	n.d.	n.d.	n.d.

[a] *n*=5, the value presents the 5 first fractions of the 0.5 % cyclic extraction. [b] HK=Hibbert's ketone. [c] n.d=not detected.

Interestingly, strong signals were observed at 6.7/112.5 ppm and 6.6/120.5 ppm, typical of C2Ar–H and C6Ar–H correlations, respectively, in 5–5′ condensed subunits.^32^ This is further substantiated by HMBC (Figure S27). These signals were not as intense in milled wood lignins (MWL) prepared from the original wood and the fibers after subcritical water extraction (fiber residue A, Figure 1; for HSQC spectra, see Figures S24 and S25). This suggested that some lignin condensation reactions occurred during the 2 h and 3 h organosolv extractions. In contrast, signals from noncondensed C5Ar structures were relatively weak in the organosolv lignins. These structures are analyzed indirectly by the drift they cause to C2Ar–H and C6Ar–H chemical shifts, which then appear at 6.92/110.5 ppm and 6.8/118.5 ppm, respectively. The formation of 5–5′ structures cannot occur through acid‐catalyzed condensation under the prevailing conditions since the free aromatic sites are known to be electron‐rich. A mechanistic pathway leading to their formation is discussed in a later section.

### Extraction trend investigation and method development of the cyclic extraction method

**Figure 5:**
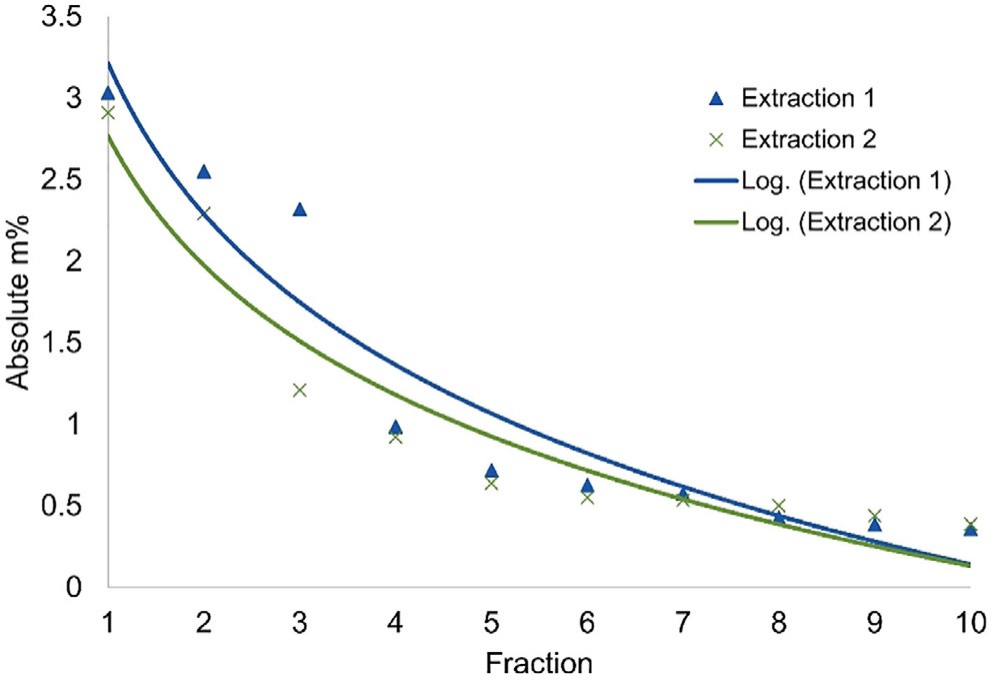
Yield trend for cyclic extraction using 0.5 wt % acid.

**Figure 6:**
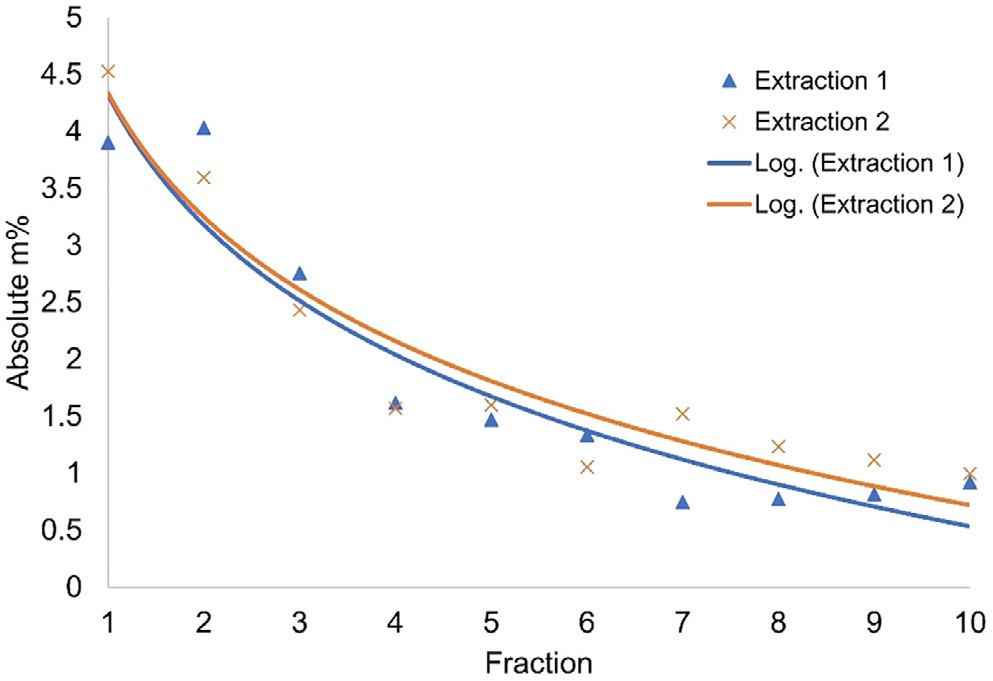
Yield trend for cyclic extraction using 1.5 wt % acid.

Next, we investigated a physical protection strategy with the expectation of better preserving the native lignin interunit linkages. Hence cyclic extraction was explored. Here, static cycles consisting of 5 min aqueous ethanol extractions at 160 °C, using two acid concentrations (0.5 and 1.5 wt %), were performed on the subcritical water‐extracted fiber residue. The obtained lignins were analyzed for yield, hydroxy functionality (using ^31^P NMR spectroscopy), lignin structure (using 2 D NMR techniques) and molecular weight distribution (using SEC). Yield analysis showed that the highest quantity of lignin was extracted during the earlier cycles (Figures 5 and 6) with a steep decline in efficiency from cycle 1 to 4. When compared to the longer 2 h reference extractions described earlier, the yields (56.3 %, Table 1) are in the same regime, indicating that the kinetics of lignin extraction are improved when using the cyclic method. This might be explained by saturation being impeded by the periodical exchange of solution for fresh solvent, which enhances the extraction.

**Figure 7:**
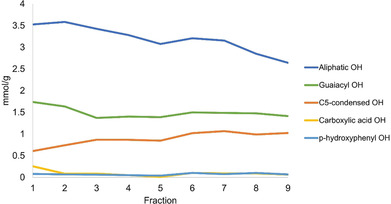
^31^P NMR spectroscopy: Extraction trends in static cycles using 1.5 wt % acid.

The results of ^31^P NMR spectroscopy (Figure 7 and Table S3), show a relatively high content of aliphatic hydroxy groups in the earlier part of the cycle, suggesting that the native aliphatic side‐chain configuration is quite well preserved. In native wood lignins, Cα and Cγ are mostly hydroxylated, the latter to a higher degree than the former. The content of aliphatic hydroxy groups seems to decrease with increasing cycle number, indicating side‐chain reactions. The content of noncondensed phenolic hydroxy groups decreases slightly and then seems to level off as the cycle number increases. This suggests that lignin depolymerization, which normally occurs through aryl ether cleavage, is not significant when using the static cycle method. In contrast, the content of C5‐condensed phenolic hydroxy groups increases slightly at the beginning of the cycle then level off.

**Figure 8:**
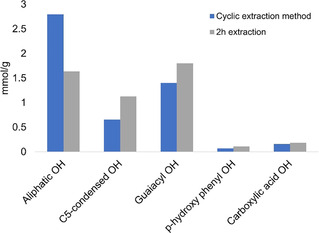
Comparison of hydroxy functionalities between the integrated cyclic extraction method (1.5 % acid series) and the 2 h organosolv extraction method.

A comparison between the hydroxy functionalities of the lignin obtained by the integrated (average values) cyclic extraction method and that obtained from the 2 h organosolv extraction is shown in Figure 8. The 2 h extracted lignin is found to have a lower aliphatic hydroxy content and a higher phenolic hydroxy content; these observations are consistent with the occurrence of side‐chain reactions and the cleavage of aryl ether linkages, respectively.

**Figure 9:**
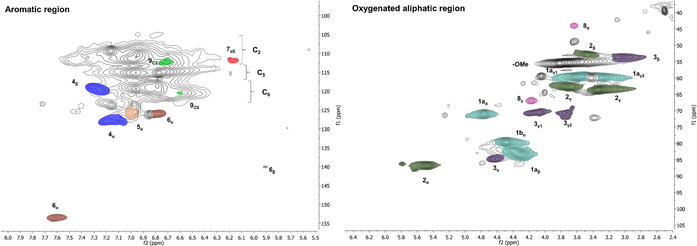
HSQC spectra for integrated cyclic extraction method. f1: ^13^C, f2: ^1^H. The figures are colored and labeled according to the structures depicted in Figure 3.

The native interunit linkages (Figure S2) in the pooled cyclic extracted lignins (together with other linkages; see Figure 3) were investigated by HSQC (Figure 9). The previously discussed signals relating to 5–5′ condensation products—C2Ar–H and C6Ar–H appearing at 6.7/112.5 ppm and 6.6/120.5 ppm, respectively—were compared between the cyclic series (Figure 9, for full HSQC spectrum, see Figure S20) and the reference samples (Figures S18 and S19). Stronger signals in support of the condensation reaction could be observed in the reference samples, consistent with the ^31^P NMR results (Figure 8). In addition, β‐O‐4′ bonds are present in both α‐hydroxylated and etherified forms.

**Figure 10:**
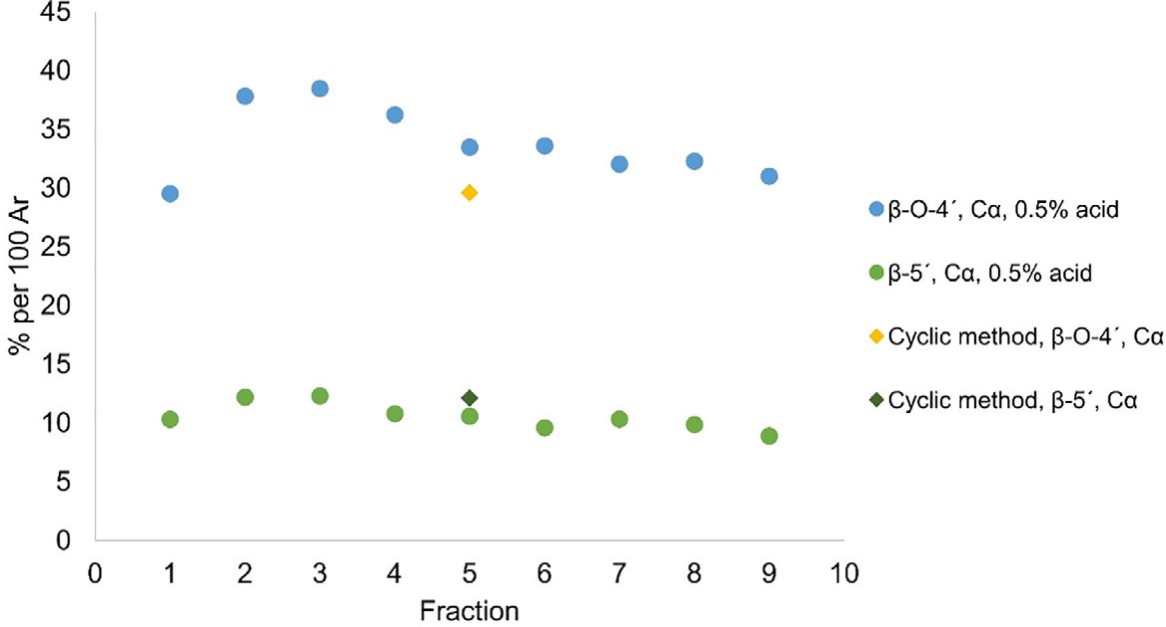
Trend of the interunit linkage content of β‐O‐4′ and β‐5′ interpreted from HSQC. Cα indicates the alpha carbon on the benzylic chain of the lignin molecule, both with hydroxy and ethyl substitution.

The trends of β‐O‐4′ and β‐5′ concentrations from the 0.5 % acid study and the final cyclic method are compared in Figure 10. From fractions 2 to 9 in the trend study, β‐O‐4′ concentrations are seen to decrease slightly, but are still in the region of 30–38 per 100 aromatic rings, and are significantly higher compared to the aforementioned organosolv reference with around 7 per 100 aromatic rings. In fact, the β‐O‐4′ amounts in the cyclic series are at similar levels to those of milled wood lignins (MWL) prepared from both the original wood and the subcritical water‐extracted wood meals used in this study. High yields of lignin with good preservation of native structures using static cycles can thus be substantiated. The mildness of the extraction is also manifested in the detection of dibenzodioxocin and trace amounts of spirodienone structures (Figure S17), which are traditionally easily modified or degraded during extraction.

**Figure 11:**
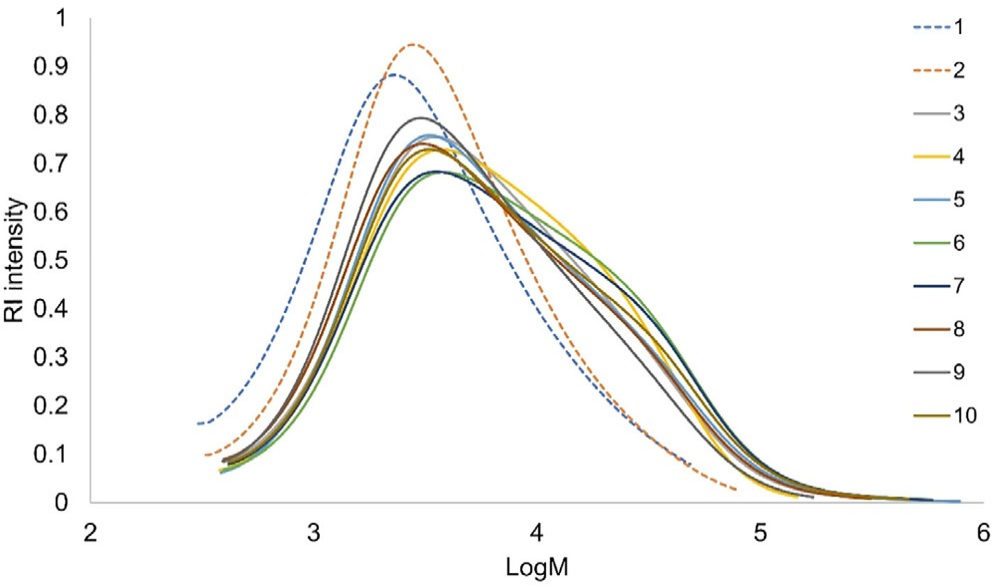
Trends in size‐exclusion chromatography using 1.5 wt % acid, fractions 1–10.

SEC was conducted on the cyclic fractions (Figure 11 and Table S5). It is seen from the chromatograms that the lignin from the first two cycles have a single distribution while the lignins from the third cycle and upwards show overlapping chromatograms. The polydispersity index *Đ* is 3.0–4.8 and the DP_n_ is 9–19 for the ten cyclic fractions. Except for the first three cycles, there is no linear trend in molecular weight (Table S5).

**Figure 12:**
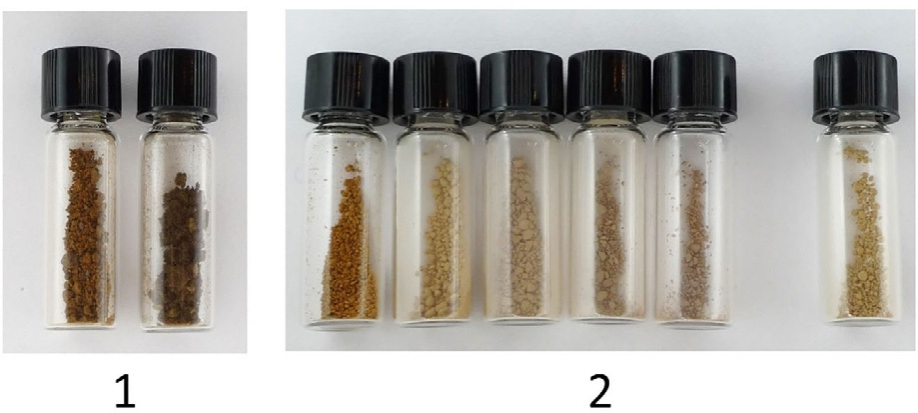
Photographs of lignin fractions. 1) Reference extraction of 2 h and 3 h, respectively. 2) 1.5 wt % acid extraction series of ten fractions; left to right: fractions 1–5 and fraction 10. The images are taken with white copy paper as the background.

From ocular inspection, we observed that the lignin fractions have different colors and that the more “native”‐like lignin fractions obtained by the cyclic method appear paler, with a light beige color, compared to the reference organosolv lignins extracted at 2 h and 3 h, which have a significantly darker color (Figure 12). This difference in color is probably due to a higher degree of lignin condensation in the reference lignins. Interestingly, the first sample in the cyclic lignins sticks out in being slightly darker than the rest. This could be explained by the presence of extractives in this first cycle. From the HSQC (Figure S23), signals from extractives were more prominent in this fraction than the subsequent ones. These signals appear in the nonoxygenated aliphatic regions. HMBC (Figure S26) analysis substantiated the presence of unsaturated fatty acids or esters. The 0.5 % acid series fractions followed the same color patterns as the 1.5 % series (Figure S32).

For practical reasons, the cyclic method was further developed as an integrated method, that is, all cycles were pooled. For this purpose, the 1.5 % acid series was chosen over the 0.5 % acid series. This was due to the higher lignin yield, although the β‐O‐4′ content was slightly lower, Table 1. The final integrated cyclic method (Figure 9) reflects an average of interunit linkages from all previous cycle series with a β‐O‐4′ content of 30 % and a β‐5′ content of 12 % (Table 1). The collected results from the different extraction series, based on the consolidated biorefinery steps illustrated in Figure 1, are given in Table S10.

### Fractionation for narrow polydispersity

The sample obtained from the integrated cyclic method was studied by SEC and shown to have a DP_n_ of around 7 and *Đ* of 4.4 (Table 2). In general, low *Đ* values are preferred if such lignins are to be used directly as polymer precursors. The crude fractions obtained here could require further refining due to their high *Đ* values.

**Table 2:** Fractionation of cyclic extracted lignin. The yield is given as relative mass % to the total amount of extracted lignin.

Fraction	*Đ*	DP_n_	Yield [%]
Cyclic method	4.46	7	100
EtOH soluble	2.15	4	48
EtOH insoluble	3.68	13	52

To decrease *Đ*, fractionation with ethanol (99 %) was investigated and found to be efficient. Two fractions were obtained: an ethanol‐soluble fraction and an ethanol‐insoluble fraction, with yields of 48 % and 52 %, respectively, and *Đ* values of 2.1 and 3.6, respectively (Table 2). The insoluble fraction had a higher molecular weight than the soluble fraction, yet, interestingly, both fractions had a similar content of β‐O‐4′ bonds, at 30 % (Figures S21 and S22). This observation has mechanistic implications and will be discussed later. Details regarding the SEC analysis are given in Figure S29 and Table S7.

^31^P NMR spectroscopy (Table S4) show that the C5‐condensed phenolic content is lower for the ethanol‐soluble fraction than for the insoluble fraction, as well as for the unfractionated lignin sample. Fractions 1 and 2 from the 1.5 % acid cyclic series were studied in a similar fashion. In both cases, the initial *Đ* was reduced for the ethanol‐soluble fractions. A similar trend was observed for fraction 2 (Figure S28 and Table S6). Overall, narrow *Đ* values were successfully obtained by post‐fractionation using ethanol as a sustainable solvent.

### Reaction mechanisms

**Figure 13:**
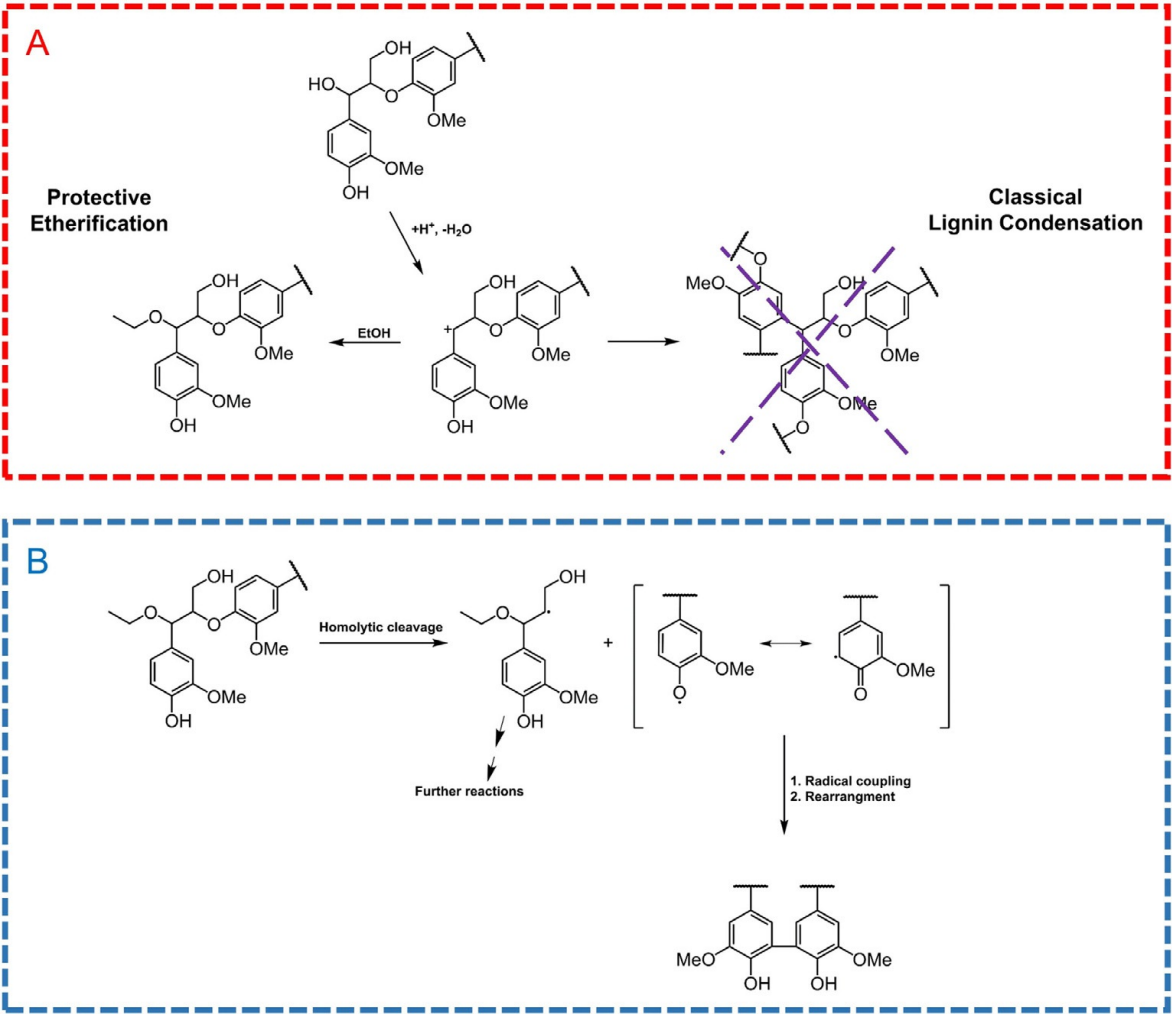
A) Lignin reactions during static cycle extraction. B) Lignin reactions during prolonged batch extraction.

From the results presented so far, a few points stand out to reveal insights into a mechanistic understanding of the extraction process. These will now be discussed. When comparing the lignins obtained through short cycles with the reference (2 h) extractions, it is observed that the degrees of Cα etherification in β‐O‐4′ structures are similar (60–65 % etherified, whereas the rest are hydroxylated). In the 3 h reference, by contrast, the degree of Cα etherification in β‐O‐4′ structures is almost 100 %. These observations suggest that etherification takes place continually under acidic conditions and that the formed ethers are stable. Interestingly, the β‐O‐4′ contents are significantly different (30–38 % levels in cyclic method compared with 4–7 % in references), suggesting that Cα etherification does not protect the β‐O‐4′ structures from cleavage reactions. On the contrary, etherification may provide chemical protection from lignin condensation reactions by means of capping the benzylic cation with ethanol. β‐O‐4′ structures are physically protected through the developed cyclic extraction processing strategy. The physical protection results from the periodic removal of the dissolved components from the reactor to ambient conditions. In this way, the dissolved molecules are not exposed to the reaction conditions for a long duration, thereby limiting further reactions. The lignin reactions that do take place during the short residence time in the cycle are shown in Figure 13 A. In addition, the solute concentration is maintained at a low level, owing to displacement with fresh solvent. This results in a lower probability of occurrence of lignin condensation reactions, which would require molecular collisions.

Analysis of the number average molar mass (*M*_n_) of the lignin fractions obtained by the cyclic methods suggests some differences, yet the β‐O‐4′ contents of these fractions are the same. A similar observation can be made from the lignin that was refined further by ethanol fractionation (ethanol‐soluble and ethanol‐insoluble fractions, Figures S21 and S22). In fact, analysis of the DP_n_ of these refined fractions (Table 2) shows a threefold difference. This suggests that condensation reactions between fractions with similar content of β‐O‐4′ structures take place. The content of β‐O‐4′ structures in the formed molecule resulting from such condensations would be the same. Several types of condensation reactions could potentially occur under acidic conditions. HSQC analysis showed the presence of stilbene structures, which are formed from the elimination of formaldehyde in phenylcoumaran (β‐5′) and spirodienone (β‐1′) structures. This formaldehyde could participate in condensation reactions involving the electron‐rich position *para* to the methoxy group. However, no signals in the HSQC spectra that would attest to the formation of a methylene bridge in the resultant product could be identified. On the other hand, the 5–5′ couplings showed a stronger signal in the reference organosolv lignins when compared to milled wood lignins (MWL) from the original wood meal and the subcritical water extracted residues. This indicated that these bonds were also formed during the organosolv conditions. We propose a mechanism for how these are formed. This is indicated in Figure 13 B. Prolonged exposure of the dissolved lignin to the high extraction temperature induced homolytic cleavage of some labile β‐O‐4′ linkages resulting in the simultaneous formation of beta radicals and phenoxy radicals. The phenoxy radicals resonate to C5 radical structures, which couple to form stable 5–5′ linkages.

Apart from the 5–5′ couplings seen in the HSQC analysis, the formation of C5‐condensed phenolics is further supported by ^31^P NMR spectroscopy for the cyclic extracted lignin samples (Figure 7), where an increasing trend is observed. Substantiation of the proposed mechanism is even more clearly consistent with our observations that the lignins obtained through the static cycle approach have a significantly higher β‐O‐4′ content and lower content of 5–5′ structures, when compared to the 2 h and 3 h extractions.

Another possible reaction is the cleavage of β‐O‐4′ structures by heterolysis, but this would result in the formation of Hibbert's ketones, which were only detected in small amounts by the HSQC analysis (Table 1). Homolysis therefore seems to be the main reaction pathway for the cleavage of β‐O‐4′ structures, subsequently followed by radical recoupling reactions to form 5–5′ condensed structures (Figure 13). Such reactions are more prominent in the reference organosolv (2 h and 3 h extractions) and can be minimized by adopting the cyclic method approach. The temperature dependence of homolytic cleavage of β‐O‐4′ structures has been reported^33^ and the temperature used here falls in that regime (above 130 °C). The occurrence of radical repolymerization, in accordance with the described mechanism, is also supported by the SEC data (Figure S30), which shows that the 2 h‐extracted lignins are in the same molecular weight regime as the pooled cyclic extracted lignins, albeit with approximately fourfold lower β‐O‐4′ content.

### Chemical composition and mass balance of the biopolymer from the consolidated biorefinery

The analysis was performed on the Wiley‐milled wood, hydrothermal extract, and the fiber residue after hot water and organosolv extraction. The carbohydrate composition (Table S8), Klason lignin (KL; Table S9), and acid‐soluble lignin (ASL; Table S9) for spruce wood are reported in the Supporting Information. The mass balance of the extracted samples is given in Table 3. The lignin balance for the 1.5 % acid series shows that roughly 6 % of the lignin ends up in the subcritical water extract that contains the bulk of hemicellulose, 55 % of the lignin ends up in the organosolv‐obtained pure lignin fraction, and 34 % of the lignin remains in the residual fiber fraction (see Figure 1). Thus, 94 % of the lignin balance is accounted for and the remaining part is probably lost during purification as water soluble fractions for example, as lignin carbohydrates complexes. Roughly 83 % of the total wood mass balance is accounted for in the obtained fractions. The missing fractions are most likely small water‐soluble molecules that were not recovered. These include hemicellulose‐derived components, such as *O*‐acetyl groups and monomeric sugars, that result from autohydrolysis during the subcritical water extraction step and acid catalyzed hydrolysis in the subsequent organosolv extraction step. Recovery processes for such molecules will be critical for future processes.

**Table 3:** Compositions and Mass balances of the consolidated biorefinery components presented as dry mass %.

Extraction 1.5 % acid 10 fractions	Mass balance [mass %]	KL+ASL^[b]^ [mass %]	Wood lignin [mass %]
Spruce wood	95.0	31.4^[a]^	–
HW extract	16.6	12.0	6.34
Fiber residue	45.5	23.1	33.5
Lignin extracted^[a]^	17.1	100	54.5
Total	–	–	94.4

[a] Including extractives. [b] KL=Klason lignin; ASL=acid‐soluble lignin.

### Potential uses of components from the consolidated biorefinery

The hemicellulose‐rich extracts could be hydrolyzed to monomeric sugars for further production of platform chemicals. They could also be fermented to ethanol, which would not only be attractive for the organosolv process economics but could also support the circularity of the process if the produced ethanol was used internally. The cellulose‐rich fiber fraction could also be used in a similar way to the hemicellulose fractions described above. Alternatively, fiber‐based composites are becoming attractive and the presence of lignin in the fibers has been shown to enhance the thermomechanical properties of such materials.^34^ Based on the lignin content of the fiber residue in this work, such potential applications could be investigated.

The lignin fraction could be used directly as a polymer precursor for material synthesis or catalytically depolymerized to platform monomers. Based on their functionality, structure, low DP_n_ and demonstrably narrow *Đ*, these lignins might be suitable as polymer precursors for the synthesis of thermosetting resins, as shown in recent studies.^35^, ^36^ In those cited studies, oligomeric fractions were shown to be preferable to polymeric fractions for the synthesis of homogeneous materials, due to their mutual solubility with other chemical components used in the synthesis.

The cyclically extracted lignin is also an attractive precursor for platform monomers. In this context, catalytic depolymerization is favored because of the high aryl ether content of the extracted lignin. In recent years, innovative methods for conversion of lignin into platform monomers have emerged and include reductive catalytic fractionation (RCF)^37^ and base‐catalyzed depolymerization (BCD).^38^

## Conclusions

The consolidation of a lignin biorefinery with hemicellulose and fiber production using green solvents was investigated. Two solvent systems were applied in sequence, those being a subcritical water system and an ethanol–water system with the addition of acid catalysts. From these systems three fractions were obtained viz. hemicellulose, pure lignin and a fiber fraction enriched in cellulose. The study was then devoted to further investigation of the ethanol–water extraction step with the pre‐set criteria of obtaining native‐like lignins in high yield and purity. To this effect, a processing strategy to preserve the structural integrity of lignin through both physical protection and additive‐free chemical protection was developed. In this context, a static cycle extraction approach was found to be key to the fulfillment of the preset criteria thanks to the minimization of lignin condensation reactions in this setup. The static cycle method was contrasted with a classical reference ethanol–water extraction performed under the same conditions but differentiated by an unperturbed longer extraction time. Lignin condensation reactions were found to be significant in the latter method and yielded stable 5–5′ bonds. The associated condensation mechanism is proposed to start with a homolytic cleavage of aryl ether linkages forming phenoxy radicals, as well as beta radicals. The former radicals have resonance structures with radicals at position C5, which in turn can couple to form stable C−C bonds. The typically expected lignin condensations at the benzylic cation under acidic conditions did not occur, which is in part explained by a chemical protection through capping by etherification with ethanol. Furthermore, no condensation products of lignin involving formaldehyde (which is released when stilbenes are formed) could be detected.

An essential milestone for the field is the development of a fundamental understanding related to the consolidation of the lignin biorefinery. In this regard, this study provides a path towards such consolidation where green processing strategies are combined with a mechanistic understanding that is essential to optimize the processes.

## Experimental Section

### Chemicals

Wood chips were obtained from Norway spruce (*Picea abies*). The water used in the experiment was in all cases Milli‐Q water (Millipore, Q‐POD, Millipak 0.22 μm filter). Ethanol (absolute) and acetone were purchased from VWR chemicals, sulfuric acid (>95 %, analytical grade) from Fischer chemicals, and lithium bromide (reagent grade, ≥99 %) from Honeywell. Dimethyl sulfoxide (anhydrous, ≥99.9 %), [D_6_]DMSO (99.9 at. % D), *N*,*N*‐dimethylformamide (anhydrous, 99.8 %), pyridine (anhydrous, 99.8 %), *endo*‐*N*‐hydroxy‐5‐norbornene‐2,3‐dicarboximide (*e*HNDI; 97 %), chromium(III) acetylacetonate (Cr(acac_3_); 99.99 %), 2‐chloro‐4,4,5,5‐tetramethyl‐1,3,2‐dioxaphospholane (Cl‐TMDP; 95 %), CDCl_3_ (≥99.8 at. % D), sodium acetate (anhydrous, ≥99.0 %) and sodium hydroxide (50 % in H_2_O) were purchased from Sigma–Aldrich. Pullulan standards 342 to 708×10^3^ Da were procured from Polymer Standard Service, Mainz, Germany.

### Material

The debarked wood was milled by using a Wiley mini mill (3383‐L70, Thomas Scientific). The extraction was performed by using an ASE 350 Accelerated Solvent Extractor (Dionex, Sunnyvale, CA, USA). The samples were placed in Dionium Extraction cells, 34 mL (Stainless steel extraction cells) or 66 mL (Dionium extraction cells) in size, containing a glass fiber filter. The extract was collected in 250 mL collection bottles. Extraction filters (Duran filter funnel, diameter 60 mm, 10–16 microns) were purchased from Sigma–Aldrich. The molecular weight distribution and dispersity indices were investigated by using a size‐exclusion chromatography system using refraction index detection (SECurity 1260, Polymer Standards Service, Mainz, Germany). The system included an autosampler (G1329B), an isocratic pump (G1310B), and an RI detector (G1362A). The system was equipped with GRAM columns (Polymer Standard Service, Mainz, Germany) in a series of precolumn (10 μm, 8×50 mm), PSS GRAM 10 000 Å, and 100 Å (10 μm, 8×300 mm) columns. The data were processed by using the software PSS WinGPC UniChrom (Polymer Standard Service, Mainz, Germany). The carbohydrate analysis was performed by using an HPAEC/PAD ICS‐3000 system (Dionex, Sunnyvale, CA, USA) equipped with a CarboPac PA‐1 (Dionex, Sunnyvale, CA, USA) column (4×250 mm). The data was processed with Chromeleon 7.1 (Dionex, Sunnyvale, CA, USA). NMR spectroscopy was carried out on a Bruker NMR spectrometer 400 DMX (Bruker Corporation, Billerica, MA, USA) and Bruker NMR spectrometer Avance III HD 400 MHz (Bruker Corporation, Billerica, MA, USA). Data was analyzed by MestreNova (v.9.0.0, Mestrelab Research).

### Methods

Spruce wood chips were first debarked and ocularly examined, where only bright wood without defects was collected. The wood chips were then Wiley‐milled to 40 mesh. All the following wood meal weights are given on oven‐dry basis. Since ASE instruments are programmed to keep a certain pressure, the exact amount of liquid is not constant in the static cycles using the standard method. Another consideration is that wood components are continuously removed in the cyclic extraction, inducing a continuous change in the liquid/wood (L/W) ratio. However, the L/W ratio is still roughly estimated in the following sections presented below.

The extraction process is divided into three sections. 1) A reference sample extraction; 2) an investigation of extraction trends and properties of the lignin fractions; 3) development of a cyclic extraction method for lignin:

1) Wiley‐milled wood (3.8 g) was placed into a 34 mL stainless steel extraction cell. In the first step, a 2 h hot water extraction (HW) was performed followed by a second step comprising an organosolv extraction for 2 or 3 h. Instrument parameters were as follows: 160 °C, a fixed volume of 40 mL, and a purge time of 90 s was used for both the HW and the organosolv extraction. The extraction was performed at a pressure of 1500–1600 psi. The samples were extracted with a solvent system composed of 1.5 wt % H_2_SO_4_ in an aqueous ethanol solution (30:70 *v*/*v*). For the HW extraction, the L/W ratio was 10.5. The L/W ratio for the organosolv extraction was estimated to be 14.

2) Extraction series were made for H_2_SO_4_ additions of both 1.5 wt % and 0.5 wt % to a binary solvent aqueous ethanol solution (30:70 *v*/*v*) system. For the 1.5 wt % acid series, Wiley‐milled wood (4.80 g) was placed into a 34 mL extraction cell. A HW extraction was performed for 2 h, at 160 °C, using a fixed volume of 40 mL with a purge time of 90 s followed by an organosolv extraction which was performed 10 times for 5 min each at 160 °C, with a fixed volume of 40 mL and using a purge time of 90 s. For the HW extraction, the L/W ratio was 8, whereas that for the organosolv extraction was estimated to by 11 for the first fraction and 13 for the last. For the 0.5 wt % extraction procedure, 10.1 g of wood was placed in a 66 mL Dionium extraction cell. The parameters for the HW extraction was 2 h of extraction, 160 °C, a fixed volume of 70 mL and a purge time of 90 s. The organosolv extraction was performed 10 times for 5 min each, at 160 °C with a fixed volume of 60 mL and a purge time of 90 s. After each 5 min extraction, the extract was collected for further sample preparation. For the HW extraction, the L/W ratio was 7. The L/W ratio for the organosolv extraction was estimated to be 9 for the first fraction and 11 for the last.

3) Wiley‐milled wood (9.3 g) was placed in a 66 mL Dionium extraction cell. The amount of wood meal was linearly scaled up from the 1.5 wt % acid method [described in (2)] using 34 mL cells to the 66 mL extraction cells. First, a HW extraction of 2 h extraction at 160 °C using a fixed volume of 70 mL and a purge time of 90 s was performed. The subsequent organosolv extraction was immediately performed in 15 static cycles using the standard method, with 5 min each at 160 °C using a rinse volume of 100 % and a purge time of 90 s with the solvent system 1.5 wt % of H_2_SO_4_ in aqueous ethanol solution (30:70 *v*/*v*). In the HW extraction, a fixed volume program was used and the L/W ratio could be determined to be 8. At the beginning of the organosolv extraction, after the hemicellulose fraction had been extracted, the L/W ratio was estimated to by 10 and at the end of the cycle, it was estimated to be 12. The total amount of solvent used in the cyclic organosolv method was 340 mL.

As mentioned earlier, the ASE instrument operates at a fixed pressure of 1500–1600 psi in the standard method. When the fixed volume program was used, the solvent volume was selected to reach a pressure of 1600 psi in the cell so as to achieve sufficient pressure and subcritical conditions. The fixed volume program was used in all experiments except for the last static cycle method where the standard method was used. More liquid was used in the extraction series since every fraction was collected and analysed manually. The ASE instrument has an integrated oven and temperature control system, from which the temperature is monitored. After the system has pumped the solvent into the cell, the cell is heated for 8 min before the extraction procedure starts.

**Figure 14:**
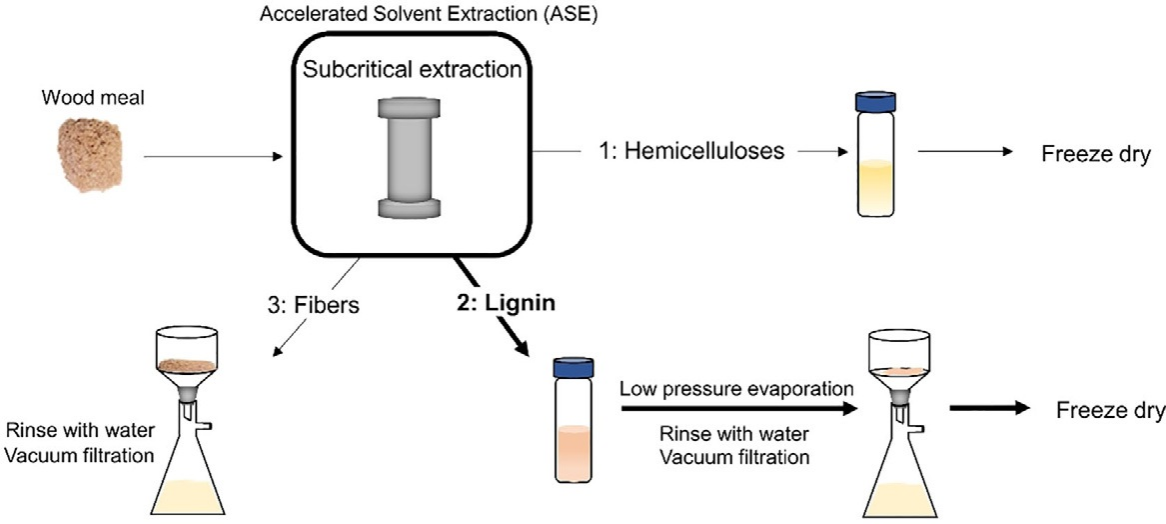
Schematic representation of the extraction method.

The HW extract was lyophilized directly. The lignin sample obtained from the organosolv extraction was evaporated under reduced pressure. During this evaporation the pH was monitored, and water was added to avoid a change in the acidity of the extract. The precipitated lignin in this acidic water solution was vacuum filtrated and rinsed with water until a clear filtrate was obtained. The efficiency of the wash was substantiated by HSQC analysis, during which no signals from carbohydrates were detected. The rinsed lignin samples were collected and lyophilized. A schematic illustration of the method is shown in Figure 14.

Fractionation was performed by adding lyophilized lignin/ethanol (1:40 *w*/*w*) to a closed vial with magnet stirring for 2 h. The solution was filtered under vacuum filtration and the residue rinsed with a small amount of ethanol.

The MWL was prepared according to the Björkman procedure^39^ with some slight modification. Shortly, in a Teflon‐lid bottle, dioxane–water mixture (200 mL, 96:4 *v*/*v*) was added to extractive‐free ball‐milled spruce wood (10 g) and the mixture was stirred at room temperature for 48 h. The dark‐brown suspension was then centrifuged (Beckman Coulter Avanti J‐E, equipped with JA 25.5 rotors at 20 000 rpm) for 20 min and the obtained supernatant was collected in a round bottom flask before being concentrated under reduced pressure to a volume of approximately 100 mL. After the addition of deionized water (150 mL) and the precipitation of the extracted lignin, the residual dioxane was completely removed by rotary evaporation. Finally, the thus‐obtained sample was freeze‐dried to obtain MWL as a light brown powder. The resulting samples were analyzed by SEC, NMR spectroscopy, XRD, and HPLC.

### Chemical composition of the biopolymer fractions

The carbohydrate composition was investigated according to the acid hydrolysis protocol.^40^ The Klason lignin and acid‐soluble lignin (ASL) contents were determined as previously reported.^41^, ^42^ Hydrolysis was performed on the Wiley‐milled wood fraction, the hydrothermal extract fraction as well as the fiber residues after hot water and organosolv extraction. In short, to 200 mg of the respective fractions, that is, wood, extracted fibers and the hydrothermal extract fractions, 72 % sulfuric acid (3 mL) was added. The mixture was placed under vacuum for 80 min with occasional stirring. The mixture was thereafter diluted with Milli‐Q water (84 mL) and placed into an autoclave for 60 min at 125 °C, following by vacuum filtration and 5×2 mL rinsing of the collected Klason lignin on the glass fiber filter.

Carbohydrate quantification was performed by using high‐performance anion‐exchange chromatography with pulsed amperometric detection (HPAEC/PAD). The method setup has been previously reported.^43^ Using 260 mm sodium hydroxide and 170 mm sodium acetate, the system was equilibrated for 7 min followed by equilibration with Milli‐Q water for 6 min. Milli‐Q water was used as an eluent at a flow rate of 1 mL min^−1^. At the column eluate, 300 mm sodium hydroxide was added before the PAD cell, at a flow rate of 0.5 mL min^−1^. Quantification was carried out by using anhydro correction factors of 0.90 and 0.88 for hexoses and pentoses, respectively, according to a previously reported method.^44^

The Klason lignin was gravimetrically quantified after being oven‐dried overnight. The ASL was quantified by UV spectroscopy at 205 nm using an extinction coefficient of 128 L g^−1^ cm^−1^ for softwood and a correction factor of 0.2 for carbohydrate degradation products.^45^

### X‐ray diffraction

The X‐ray diffraction was performed using an ARL X′TRA Powder Diffractometer (Thermo Fisher Scientific Inc., USA) using Cu_Kα_ radiation generated at 45 kV and 44 mA. The measurements were performed using scans from 2*θ*=5° to 50° in steps of 0.05° at a scan rate of 3 s per step.

### Size‐exclusion chromatography

Lyophilized sample (≈9 mg) was dissolved in a 0.5 wt % LiBr solution in DMSO (2 mL). The dissolved sample was syringe filtered using a 0.45 μm PTFE filter. SEC was run using 0.5 wt % LiBr solution in DMSO as eluent, with an injection volume of 100 μL, a flowrate of 0.5 mL min^−1^, and a column oven temperature of 60 °C. For integration, RI detection at 40 °C was used. Standard calibration was performed by using Pullulan standards in the molecular range of 342–708×10^3^ Da.

### NMR spectroscopy

For the HSQC‐edited analysis, lyophilized sample (80 mg) was dissolved in [D_6_]DMSO (600 μL). The spectra were acquired on a Bruker 400 DMX spectrometer with the “hsqcedetgp” pulse sequence using the following parameters: an acquisition time of 0.1065 s, a relaxation delay of 2.5 s, 80 scans using 1024×256 increments. Optimal pulse lengths corresponding to a 90° pulse were found for each experiment by finding and halving the pulse length corresponding to a 180° pulse where the proton FID signal was minimal. Data processing was carried out in MestReNova with 1024×1024 data points using a 90° shifted square sine‐bell apodization window. The data was Fourier transformed followed by phase correction and baseline correction in both dimensions by a Bernstein polynomial fit of order 3. Semi‐quantification of lignin interunit linkages was carried out by using the C2−H signal region on the aromatic ring as an internal standard.^46^ All NMR spectra were integrated by using the same shifts for comparable results (Table S1).

HMBC analyses were performed on the same samples as for the edited‐HSQC analyses using the same instrument and the same acquisition parameters, except for the use of the ‘hmbcgpndqf“ pulse program.

Quantitative ^31^P NMR sample preparation was performed based on a reported method.^47^ Lyophilized sample (30 mg) was dissolved in *N*,*N*‐dimethylformamide (100 μL) and pyridine (100 μL). To this solution, internal standard (IS) solution (50 μL; 60 mg mL^−1^ of *e*HNDI in pyridine with 5 mg mL^−1^ Cr(AcAc_3_) relaxing agent) was added. After stirring, Cl‐TMDP phosphorylating agent (100 μL) was added following by dropwise addition of CDCl_3_ (450 μL) to the sample solution. The ^31^P NMR spectra were acquired with 512 scans and a relaxation delay time of 6 s on a Bruker NMR spectrometer Avance III HD 400 MHz. Data processing was carried out in MestReNova. The data were Fourier transformed followed by phase correction and baseline correction in both dimensions by a Bernstein polynomial fit of order 3. Diagnostic peaks with assigned shifts are given in Table S2.

## Conflict of interest

*The authors declare no conflict of interest*.

## Supporting information

As a service to our authors and readers, this journal provides supporting information supplied by the authors. Such materials are peer reviewed and may be re‐organized for online delivery, but are not copy‐edited or typeset. Technical support issues arising from supporting information (other than missing files) should be addressed to the authors.

Supplementary
