# Supplementary material for: Toward a Consolidated Lignin Biorefinery: Preserving the Lignin Structure through Additive‐Free Protection Strategies
Source: ChemSusChem. 2020 Jun 30;13(17):4666–77. doi: 10.1002/cssc.202000974 (PMC7540675; doi:10.1002/cssc.202000974)
Supplement: Supplementary file 1 — Supplementary [file CSSC-13-4666-s001.pdf]

ChemSusChem

Supporting Information

Toward a Consolidated Lignin Biorefinery: Preserving the Lignin Structure through Additive-Free Protection Strategies

Maria Karlsson,[a] Nicola Giummarella,[a, b] Pär A. Lindén,[a] and Martin Lawoko*[a, b]

Contents

1 XRD ... 3

2 HSQC ... 3

2.1 HSQC, diagnostic peaks and structures for assignment ... 3

2.2 Common inter-unit linkages of native lignin ... 4

2.3 1.5% acid series ... 4

2.4 0.5% acid series ... 7

2.5 Mild extraction conditions ... 11

2.6 Reference spectrum 2h ... 11

2.7 Reference spectrum 3h ... 12

2.8 Integrated cyclic method ... 13

2.9 Integrated cyclic method, ethanol soluble fraction ... 13

2.10 Integrated cyclic method, ethanol insoluble fraction ... 14

2.11 Signals from extractives ... 14

2.12 Milled wood lignin (MWL) ... 15

3 ^{31}P NMR ... 16

3.1 ^{31}P NMR, diagnostic peaks for assignment ... 16

3.2 1.5% acid series ... 16

3.3 Cyclic method and ethanol fractionation ... 16

4 HMBC ... 17

5 Size Exclusion Chromatography (SEC) ... 18

5.1 Trend 1.5% acid ... 18

5.2 Fractionation of fraction 1 and 2, 1.5% acid ... 18

5.3 Fractionation cyclic extraction method ... 19

5.4 2h and cyclic extraction ... 20

5.5 Hemicellulose fraction ... 20

6 Carbohydrate analysis ... 20

7 Lignin analysis (KL and ASL) ... 21

8 Collected results 21
9 0.5% acid series samples 22

1 XRD

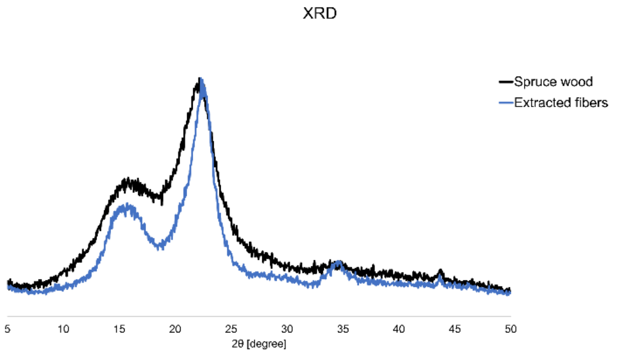

Figure S1. The XRD spectrum for spruce wood and extracted fibers. The fibers were extracted using a two-step approach in sequence of subcritical water followed by subcritical ethanol:water 70:30 (1.5% acid) using the cyclic extraction method.

2 HSQC

2.1 Diagnostic peaks and structures for assignment

All the HSQC spectra, i.e. the reference (2h and 3h), trend study of 1.5% and 0.5% acid, cyclic method and the ethanol fractionation was integrated using the same shifts for comparable results. The C2-aromatic signals were used as an internal reference.

Table S1. The diagnostic signals and integration shifts of the main interunit linkages.

HSQC	C2	β-O-4´, $C_β$	β-O-4´, $C_α$	β-O-4´, Et-$C_α$	β-5´, $C_α$
Chemical shift: ^{13}C	113.2-106.1	86.6-80.5	74.0-69.0	81.4-76.9	91.0-83.2
Chemical shift: 1H	7.8-6.1	4.6-3.9	5.1-4.4	4.7-4.0	5.8-5.0

HSQC	β- β ´, $C_α$	Stilbene, β-1´, $C_α$	Stilbene, β-5´, $C_β$	Enol ether, $C_α$	Coumarylaldehyde, $C_α$	HK, $C_γ$
Chemical shift: ^{13}C	86.4-83.1	127.8-124.4	121.8-117.8	113.7-110.4	155.5-151.7	68.5-65.3
Chemical shift: 1H	4.8-4.5	7.1-6.9	7.5-7.1	6.3-6.1	7.7-7.4	4.2-4.1

2.2 Common inter-unit linkages of native lignin

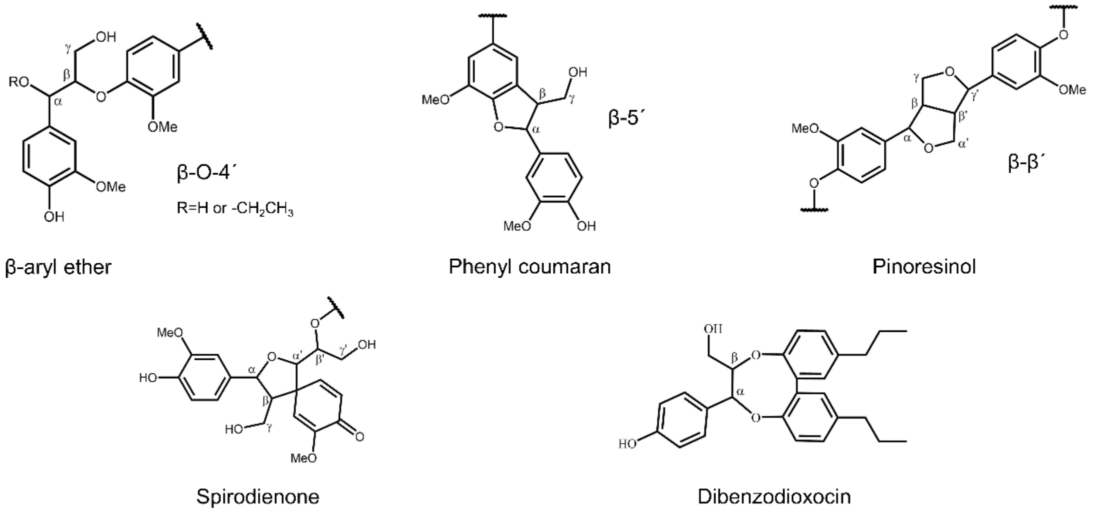

Figure S2. Common reported inter-unit linkages of lignin.

2.3 1.5% acid series

DEPT-edited HSQC NMR spectrum of the 1.5% acid series, Figure S3-S7. f1: ^{13}C, f2: ^{1}H.

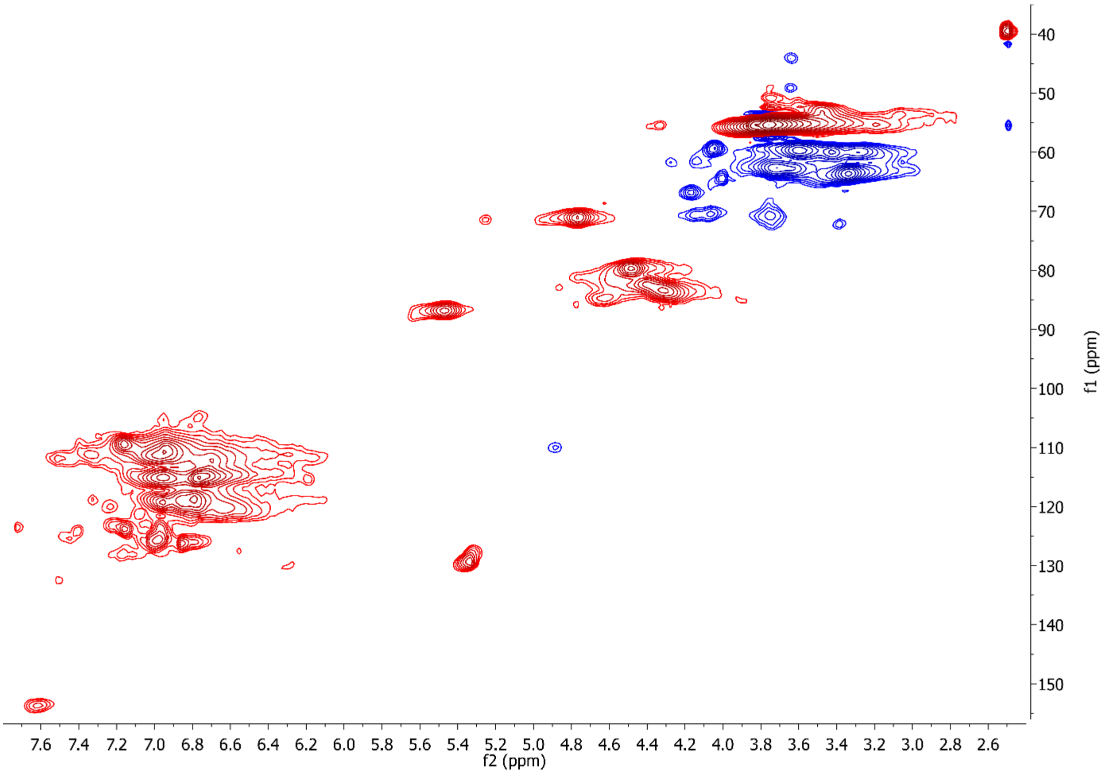

Figure S3. DEPT-Edited HSQC of Cyclic method, fraction 1, 1.5% acid. CH and CH3 signals in red, C and CH2 signals in blue.

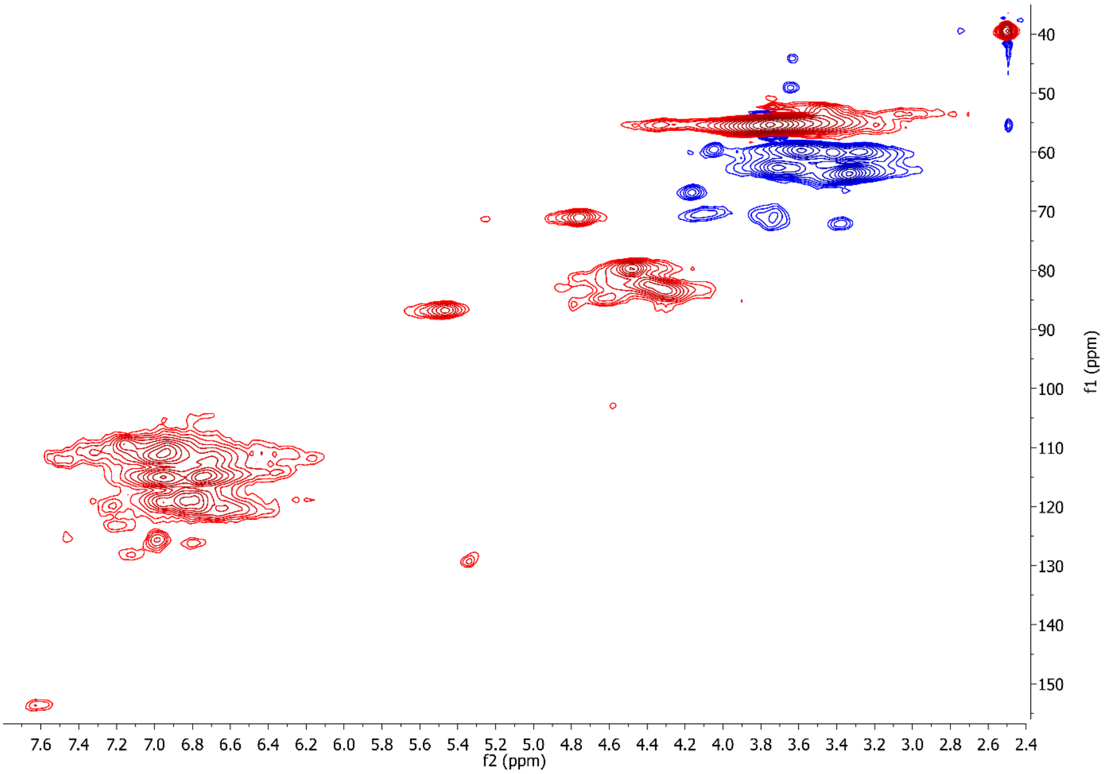

Figure S4. DEPT-Edited HSQC of Cyclic method, fraction 2, 1.5% acid. CH and CH3 signals in red, C and CH2 signals in blue.

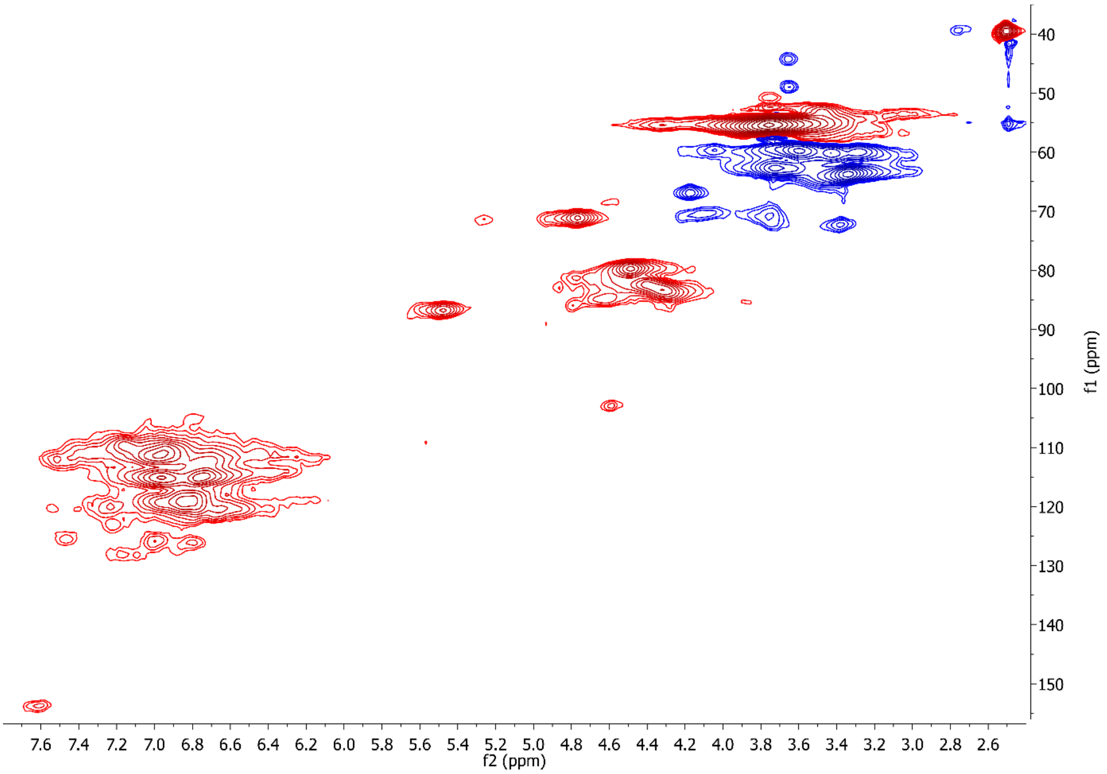

Figure S5. DEPT-Edited HSQC of Cyclic method, fraction 3, 1.5% acid. CH and CH3 signals in red, C and CH2 signals in blue.

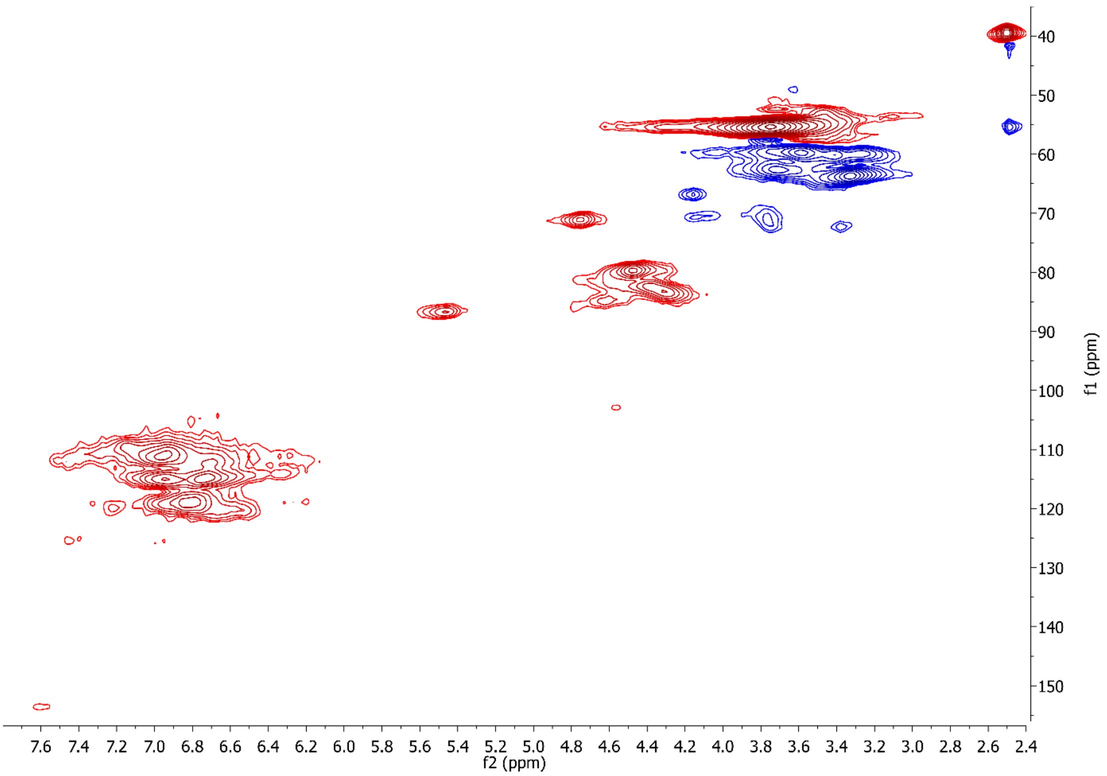

Figure S6. DEPT-Edited HSQC of Cyclic method, fraction 4, 1.5% acid. CH and CH3 signals in red, C and CH2 signals in blue.

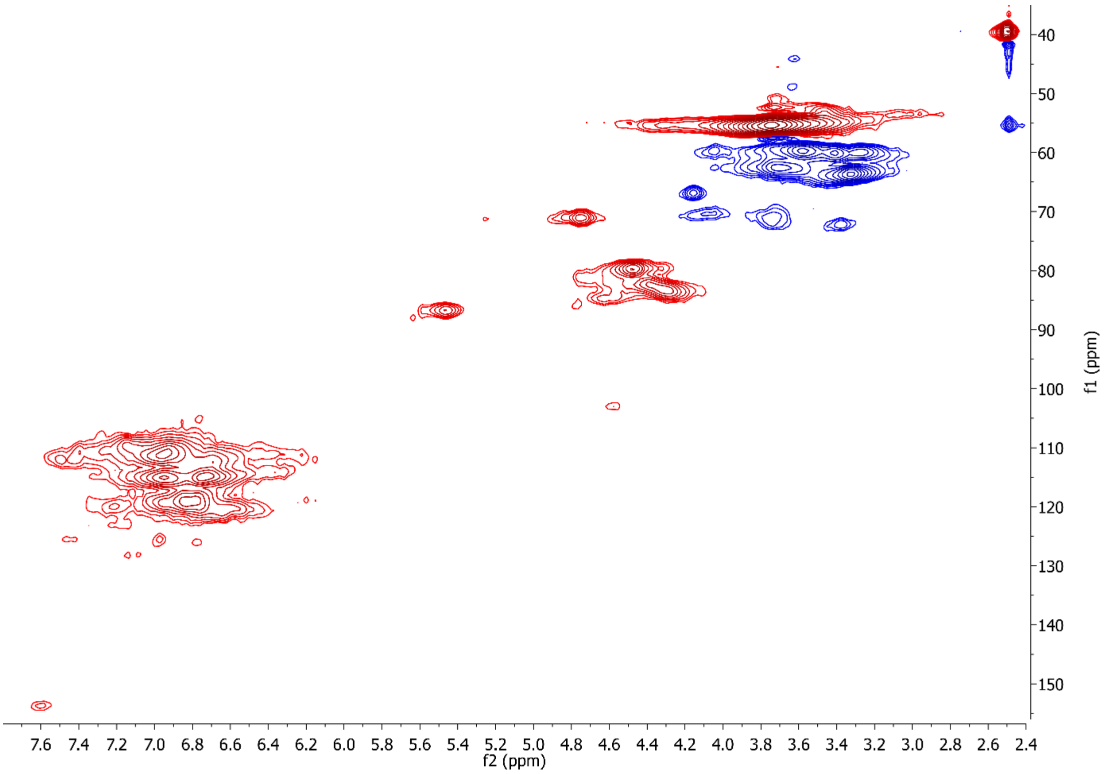

Figure S7. DEPT-Edited HSQC of Cyclic method, fraction 5, 1.5% acid. CH and CH3 signals in red, C and CH2 signals in blue.

2.4 0.5% acid series

DEPT-Edited HSQC NMR spectrum of the 0.5% acid series, Figure S8-S16. f1: ^{13}C, f2: ^{1}H.

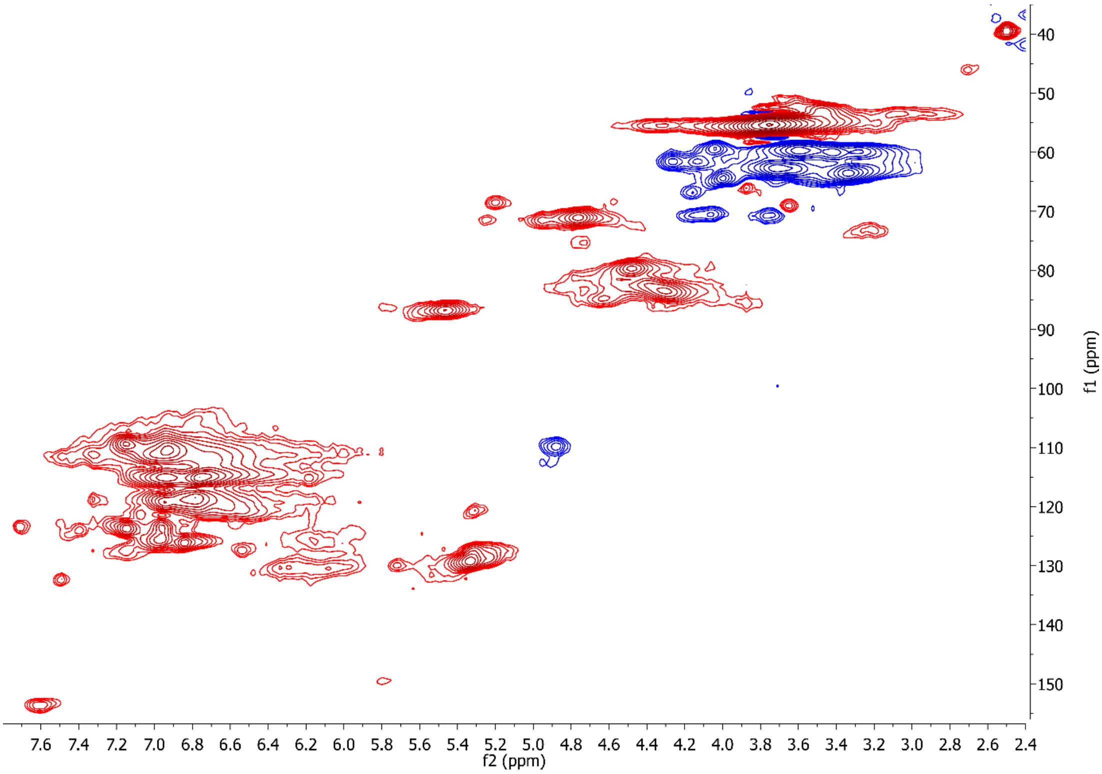

Figure S8. DEPT-Edited HSQC of cyclic method, fraction 1, 0.5% acid. CH and CH3 signals in red, C and CH2 signals in blue.

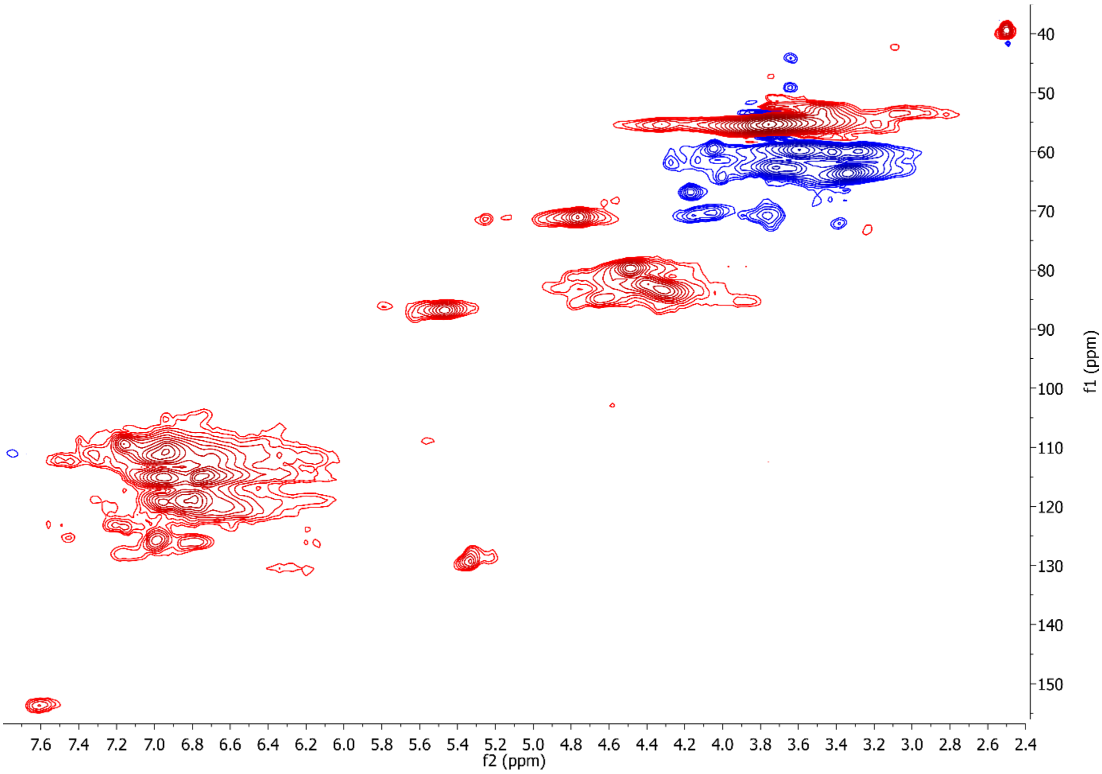

Figure S9. DEPT-Edited HSQC of cyclic method, fraction 2, 0.5% acid. CH and CH3 signals in red, C and CH2 signals in blue.

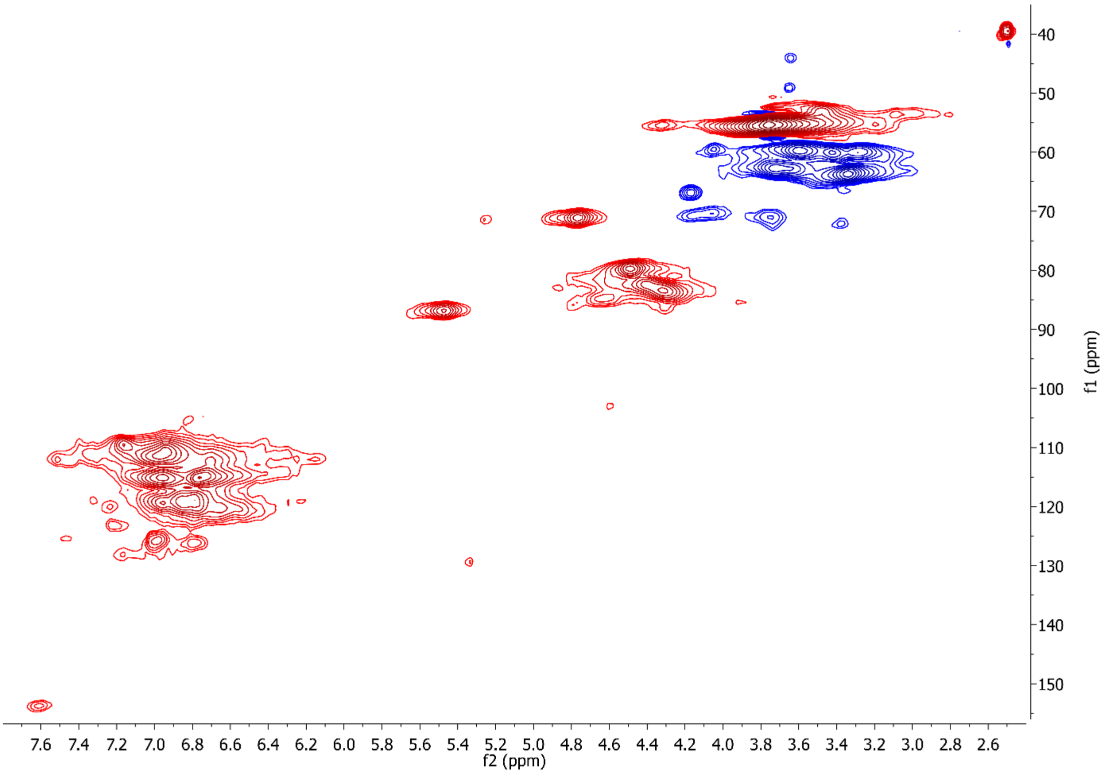

Figure S10. DEPT-Edited HSQC of Cyclic method, fraction 3, 0.5% acid. CH and CH3 signals in red, C and CH2 signals in blue.

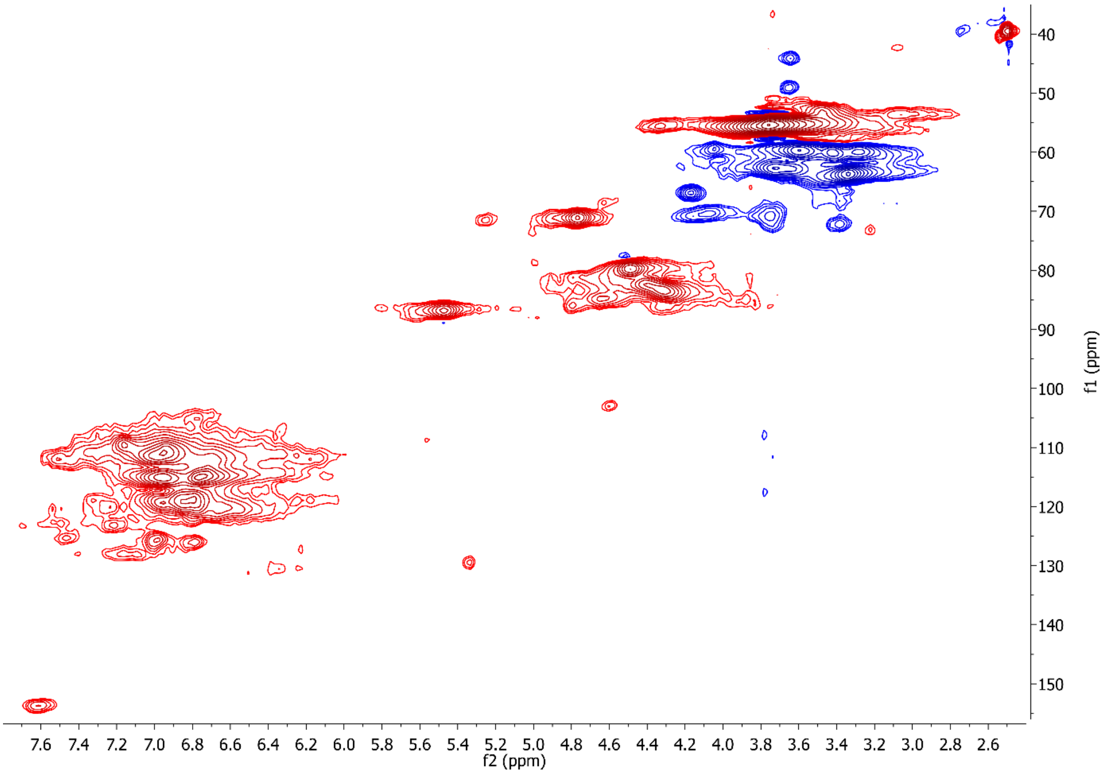

Figure S11. DEPT-Edited HSQC of Cyclic method, fraction 4, 0.5% acid. CH and CH3 signals in red, C and CH2 signals in blue.

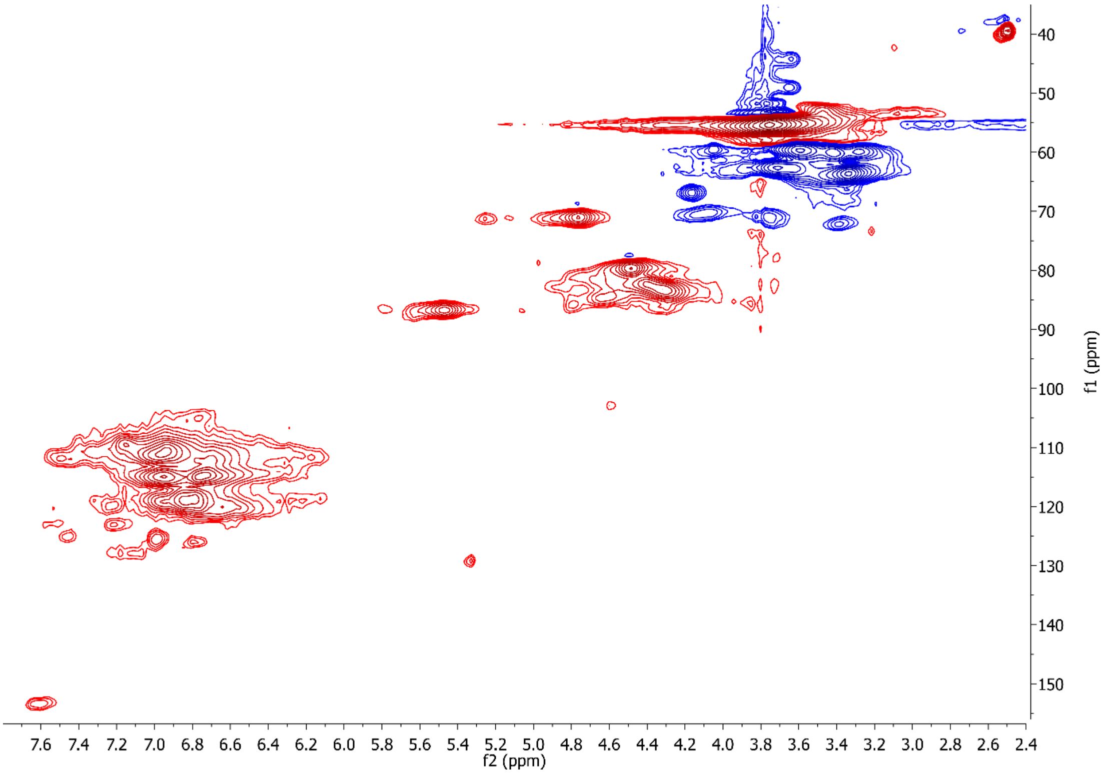

Figure S12. DEPT-Edited HSQC of Cyclic method, fraction 5, 0.5% acid. CH and CH3 signals in red, C and CH2 signals in blue.

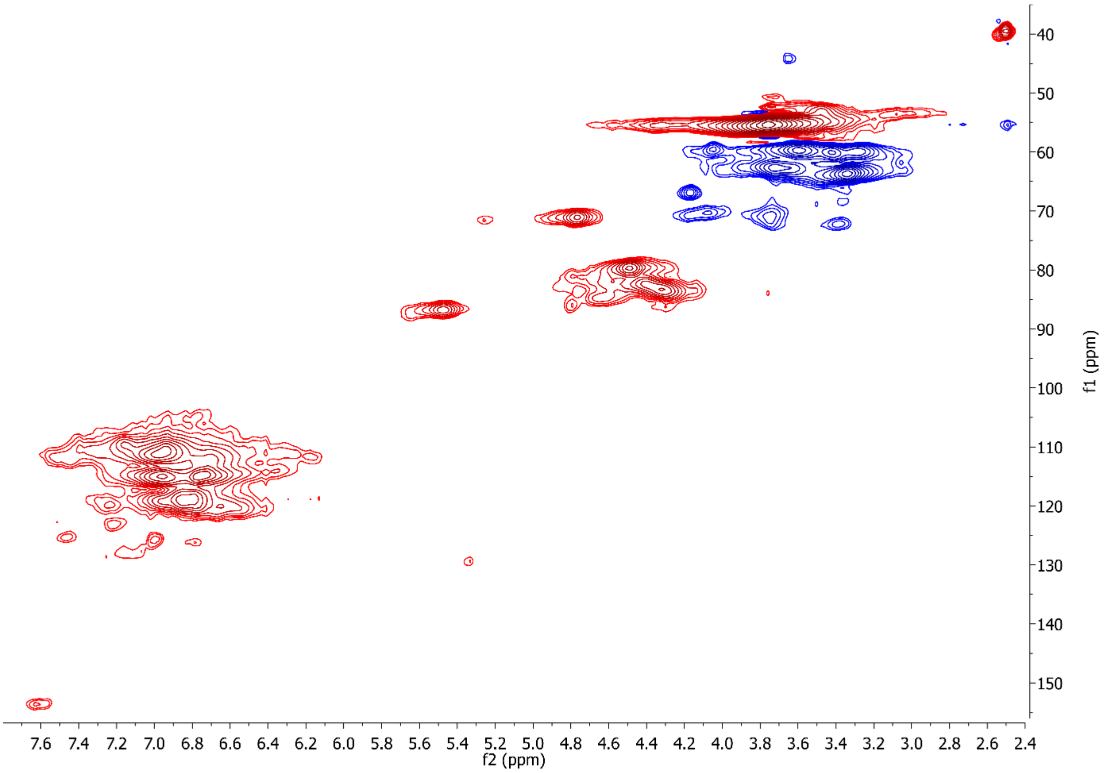

Figure S13. DEPT-Edited HSQC of Cyclic method, fraction 6, 0.5% acid. CH and CH3 signals in red, C and CH2 signals in blue.

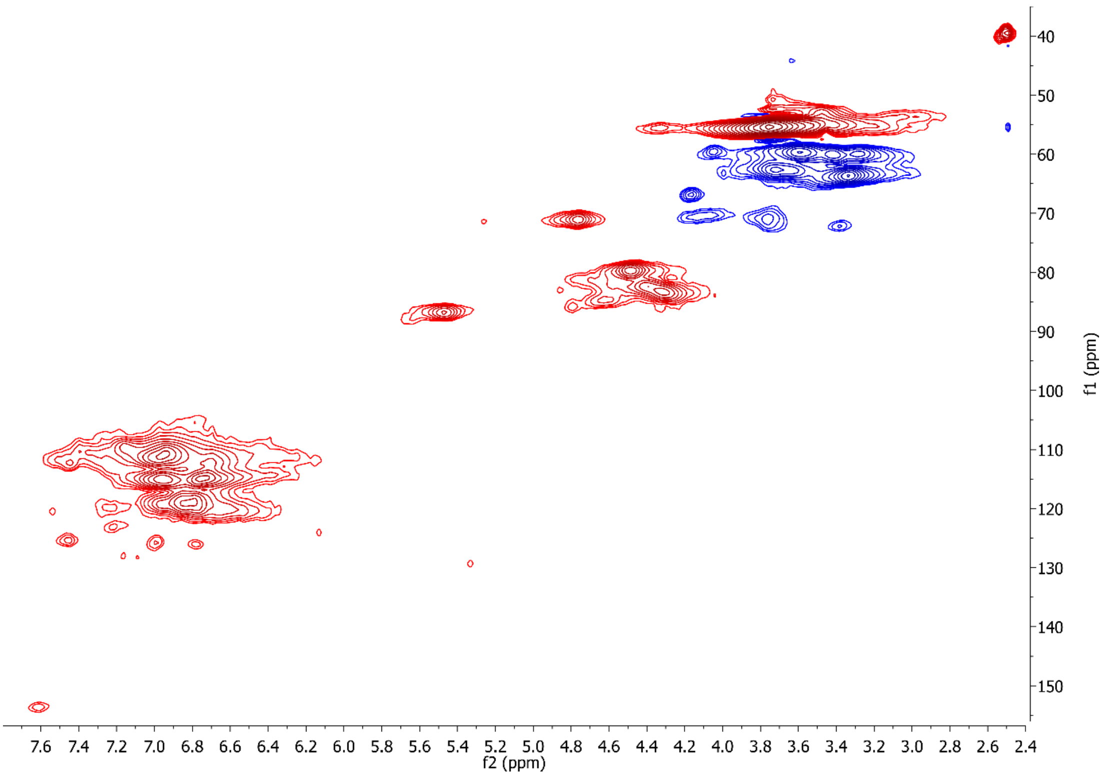

Figure S14. DEPT-Edited HSQC of Cyclic method, fraction 7, 0.5% acid. CH and CH3 signals in red, C and CH2 signals in blue.

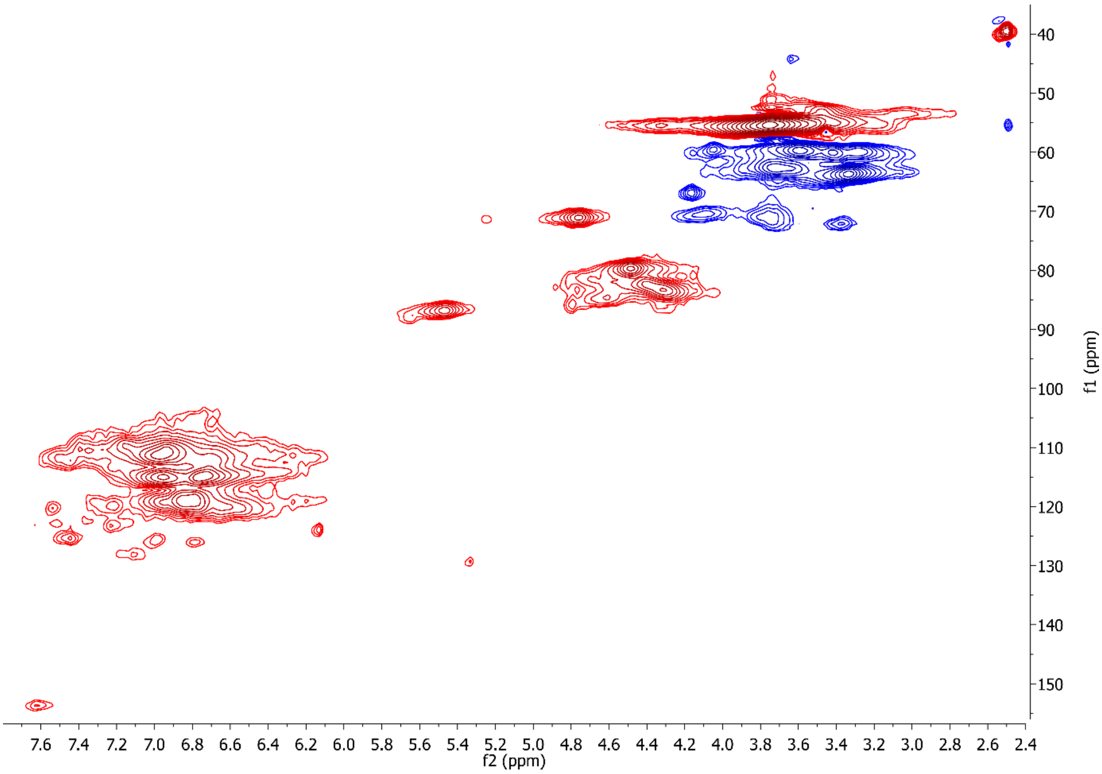

Figure S15. DEPT-Edited HSQC of Cyclic method, fraction 8, 0.5% acid. CH and CH3 signals in red, C and CH2 signals in blue.

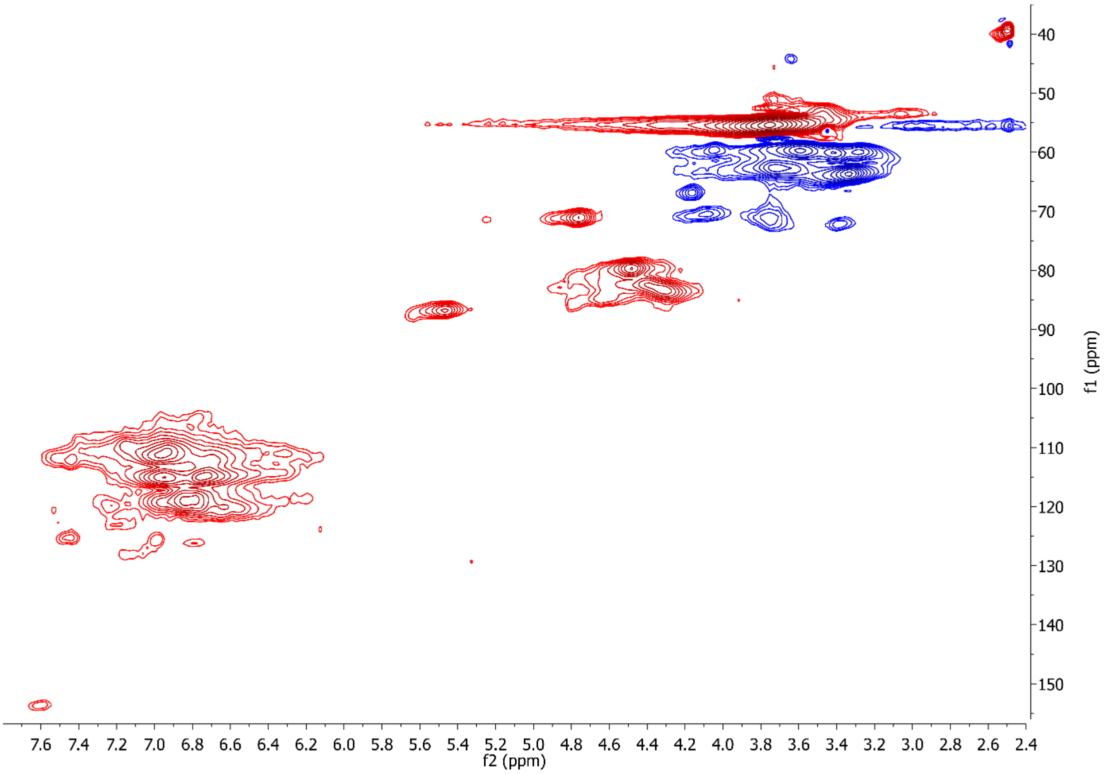

Figure S16. DEPT-Edited HSQC of Cyclic method, fraction 9 and10, 0.5% acid. CH and CH3 signals in red, C and CH2 signals in blue.

2.5 Mild extraction conditions

The signals from spirodieneone and dibenzodioxocin indicate mild extraction conditions. The HSQC spectrum in Figure S17 is from the final cyclic method using 1.5% acid.

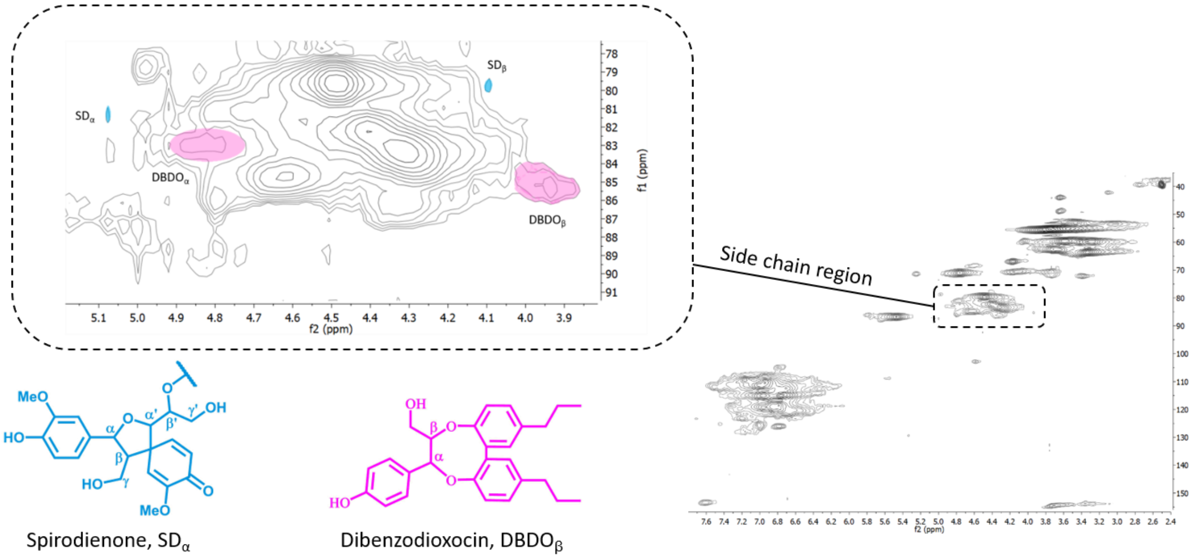

Figure S17. The signals from spirodieneone and dibenzodioxocin.

2.6 Reference spectrum 2h

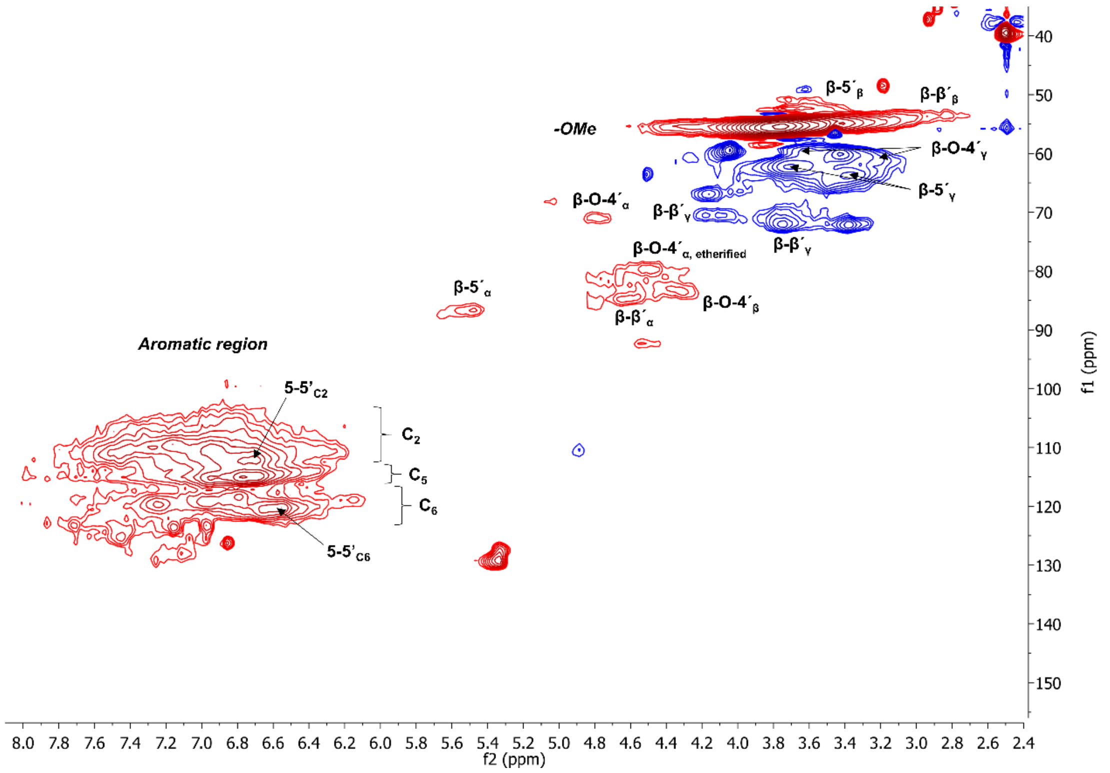

Figure S18. DEPT-Edited HSQC of reference sample, 2h extraction time, 1.5% acid. CH and CH3 signals in red, C and CH2 signals in blue. f1: ^{13}C, f2: ^{1}H.

2.7 Reference spectrum 3h

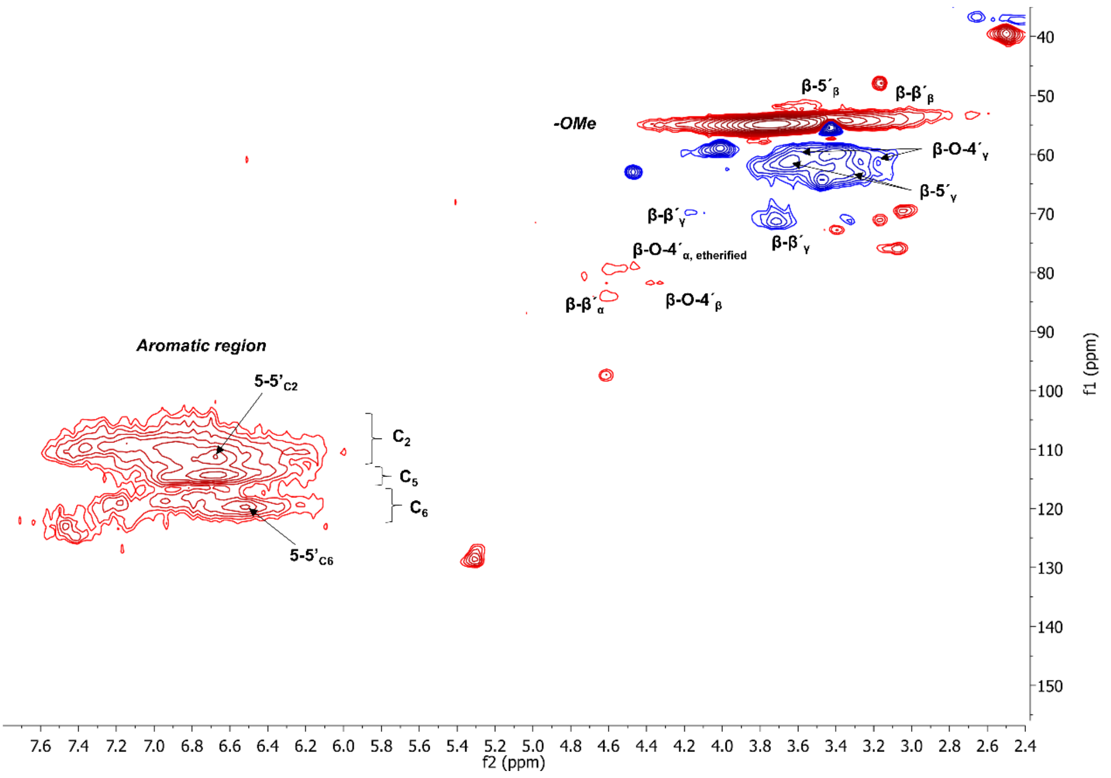

Figure S19. DEPT-Edited HSQC of reference sample, 3h extraction time, 1.5% acid. CH and CH3 signals in red, C and CH2 signals in blue. f1: ^{13}C, f2: ^{1}H.

2.8 Integrated cyclic method

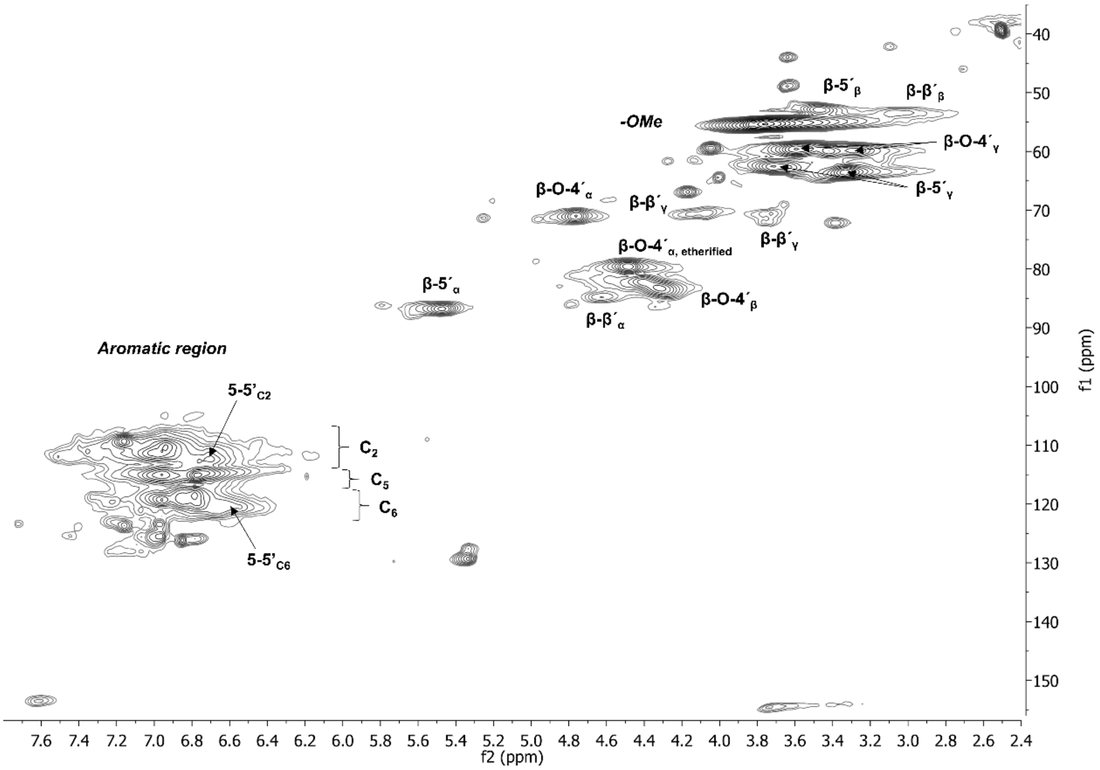

Figure S20. HSQC of the integrated cyclic method. f1: ^{13}C, f2: ^{1}H.

2.9 Integrated cyclic method, ethanol soluble fraction

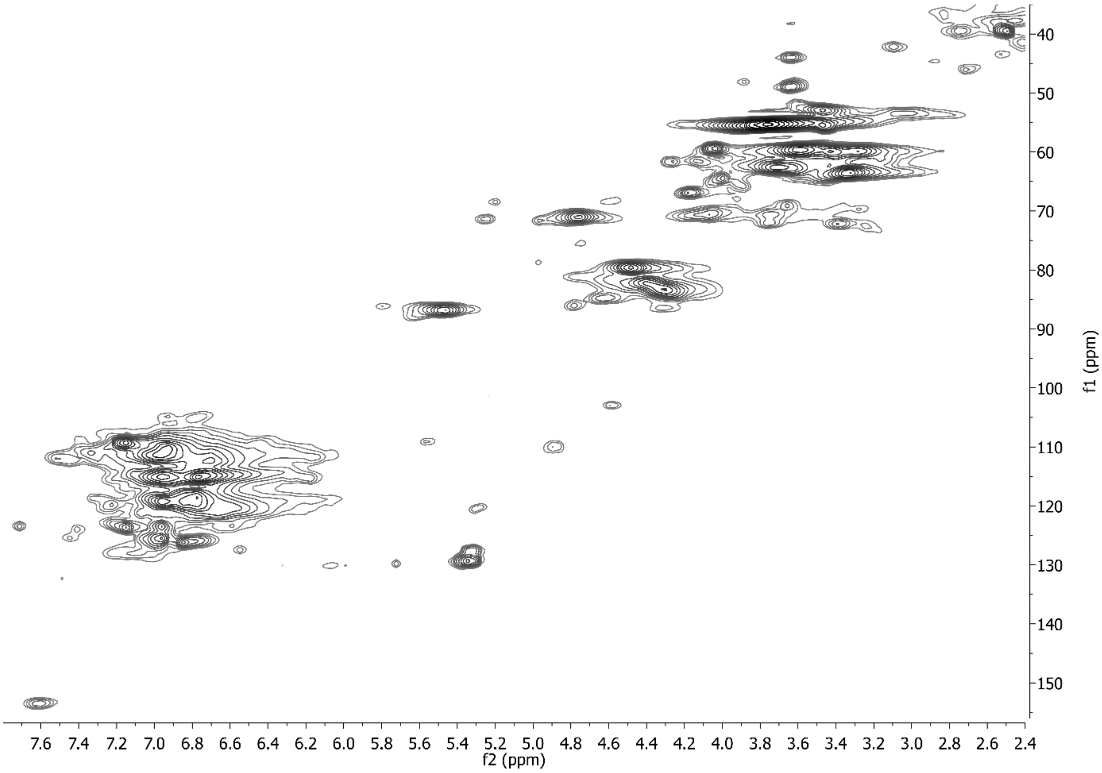

Figure S21. HSQC of integrated cyclic method, EtOH soluble. f1: ^{13}C, f2: ^{1}H.

2.10 Integrated cyclic method, ethanol insoluble fraction

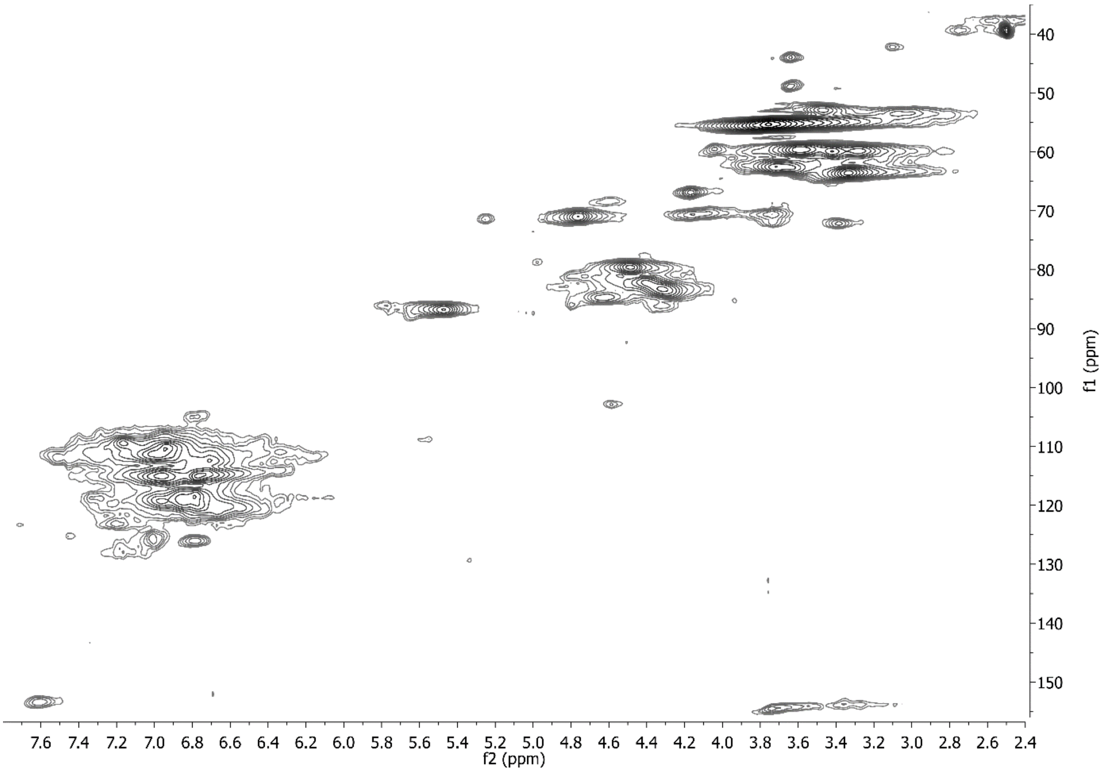

Figure S22. HSQC of the integrated cyclic method, EtOH insoluble fraction. f1: ^{13}C, f2: ^{1}H.

2.11 Signals from extractives

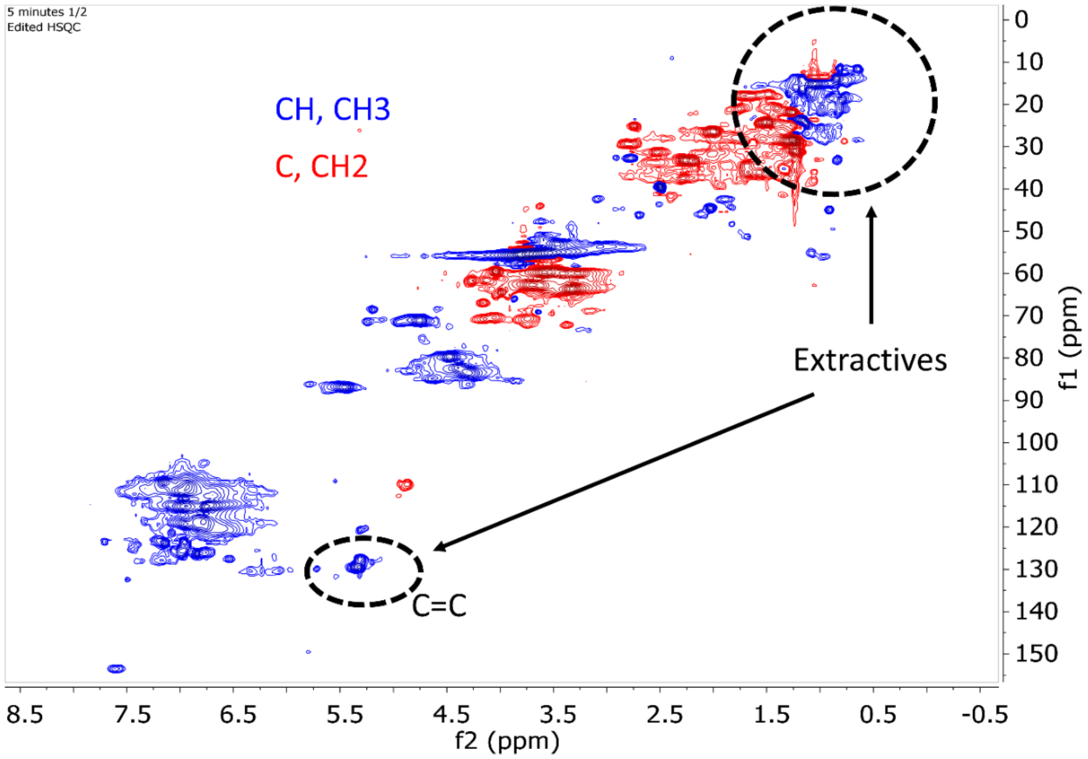

Figure S23. DEPT-Edited HSQC. Cyclic 10xfractions: Signals from extractives in fraction 1. f1: ^{13}C, f2: ^{1}H.

2.12 Milled wood lignin (MWL)

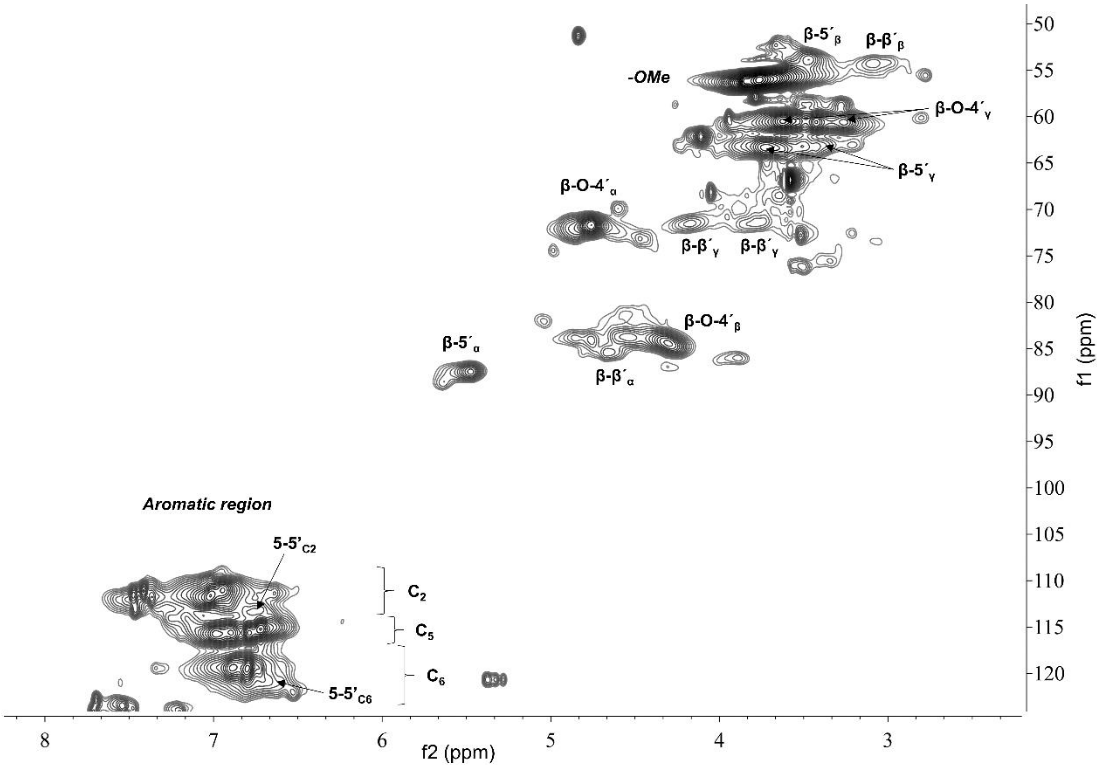

Figure S24. HSQC of MWL from wood. f1: ^{13}C, f2: ^{1}H.

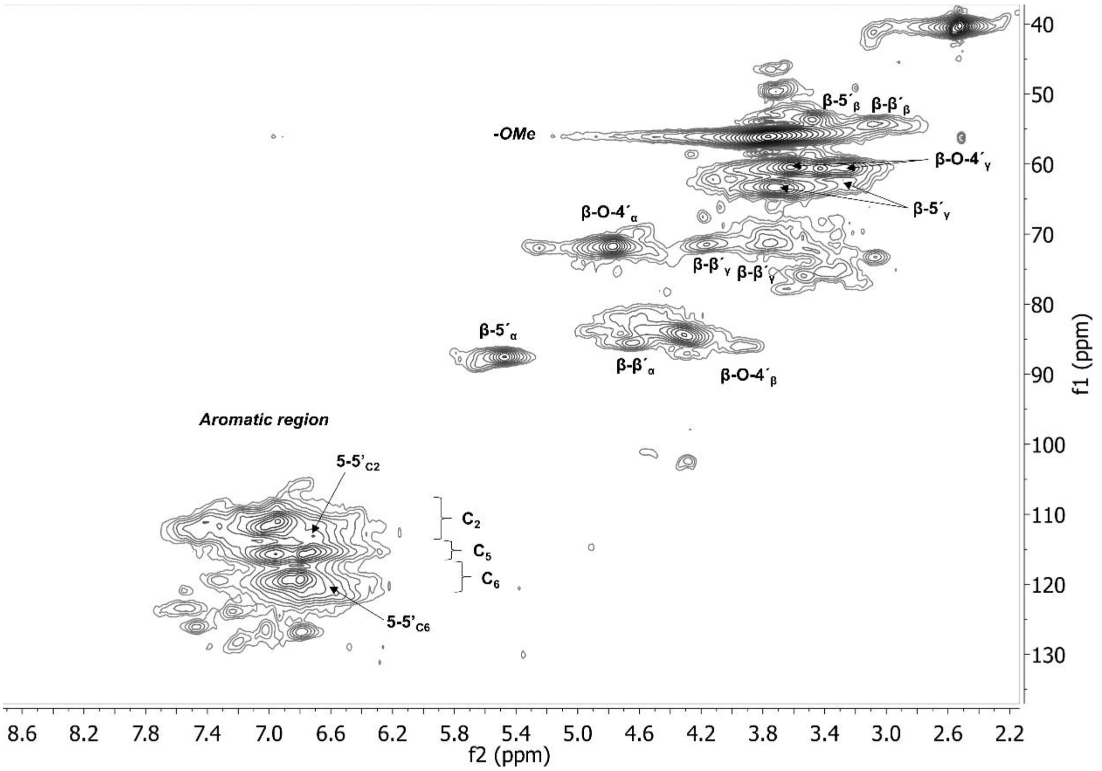

Figure S25. HSQC of MWL after hydrothermal extraction. f1: ^{13}C, f2: ^{1}H.

3 ^{31}P NMR

3.1 Diagnostic peaks for assignment

Table S2. The diagnostic peaks and integration shifts for the phosphorylated hydroxyls.

			Non-condensed phenolics		
	**Aliphatic OH**	**C_5–substituted OH**	**Guaiacyl OH**	**p-hydroxy phenyl OH**	**Carboxylic acid OH**
**Chemical shift**	149.1-145.1	144.7-141.1	140.6-138.8	138.2-137.3	136.6-133.6

3.2 1.5% acid series

Table S3. Trends of fractionated extraction of 1.5% acid series.

			Non-condensed phenolics		
	**Aliphatic OH**	**C_5–substituted OH**	**Guaiacyl OH**	***p*-hydroxy phenyl OH**	**Carboxylic acid OH**
**Fraction**	mmol/g	mmol/g	mmol/g	mmol/g	mmol/g
1	3.53	0.608	1.74	0.0795	0.256
2	3.58	0.741	1.64	0.0684	0.0855
3	3.43	0.869	1.37	0.0633	0.0863
4	3.28	0.866	1.40	0.0513	0.0513
5	3.08	0.846	1.39	0.0400	0.0114
6	3.21	1.02	1.50	0.103	0.103
7	3.16	1.07	1.49	0.0746	0.0918
8	2.85	0.988	1.48	0.102	0.0909
9	2.64	1.02	1.41	0.0686	0.0629

3.3 Cyclic method and ethanol fractionation

Table S4. Summary of the ^{31}P NMR for the cyclic method.

			Non-condensed phenolics		
	**Aliphatic OH**	**C_5–substituted OH**	**Guaiacyl OH**	***p*-hydroxy phenyl OH**	**Carboxylic acid OH**
**Fraction**	**mmol/g**	**mmol/g**	**mmol/g**	**mmol/g**	**mmol/g**
Cyclic method	2.80	0.658	1.40	0.0686	0.160
EtOH soluble*	2.97	0.595	1.62	0.0743	0.303
EtOH insoluble*	2.96	0.781	1.27	0.0849	0.119

* Fractionation of the cyclic extracted lignin

4 HMBC

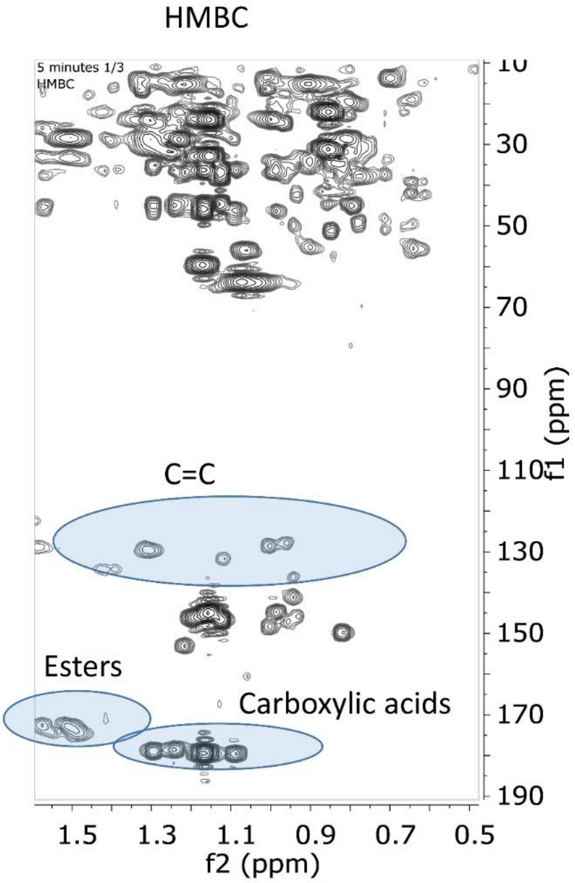

Figure S26. Presence of unsaturated fatty acids or esters.

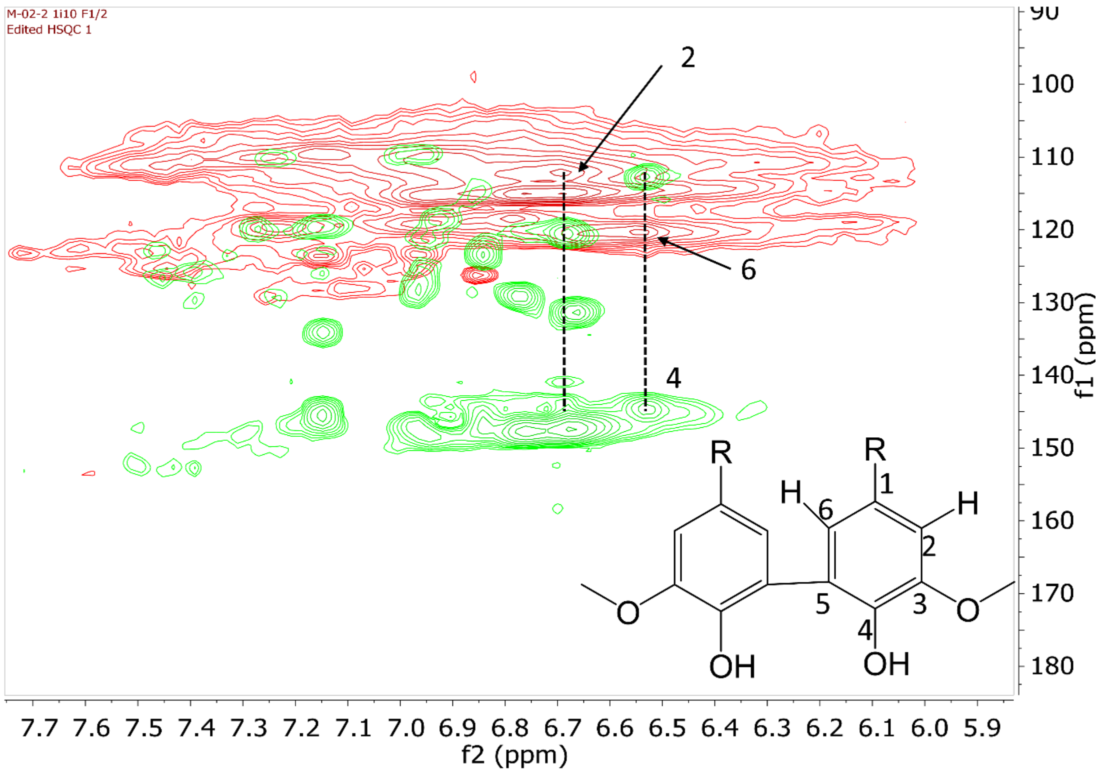

Figure S27. HSQC (in red) and HMBC (in green) overlaid spectra: strong signals at 6.7/112.5 ppm and 6.6/120.5 ppm typical of C2Ar-H and C6Ar-H correlations respectively in 5-5' condensed sub-units. f1: ^{13}C, f2: ^{1}H.

5 Size Exclusion Chromatography (SEC)

5.1 Trend 1.5% acid

Table S5. SEC data of the 10 fractions, 1.5% acid series.

Fraction	Mn [g/mol]	Mw [g/mol]	Mz [g/mol]	Đ
1	1800	5500	15100	3.04
2	2200	6500	19300	2.89
3	3100	12200	43100	3.95
4	3200	11600	32300	3.59
5	3200	14500	88300	4.57
6	3500	16600	79300	4.71
7	3400	16300	82100	4.83
8	3000	12800	53300	4.34
9	2800	10400	35500	3.71
10	3200	15000	70600	4.62

5.2 Fractionation of fraction 1 and 2, 1.5% acid

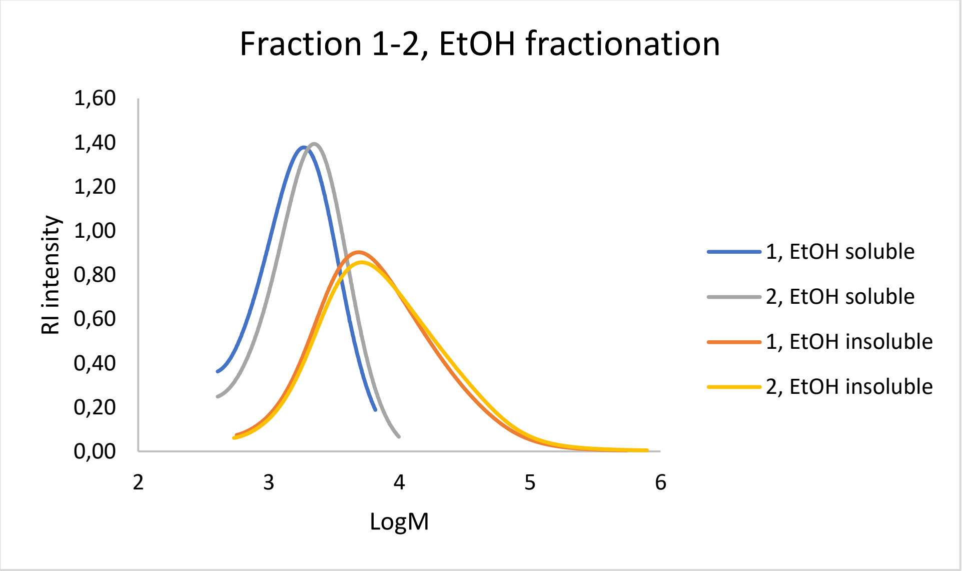

Figure S28. Ethanol fractionation of fractions 1 and 2.

SEC data for the EtOH fractionated samples.

Table S6. Ethanol fractionation of fractions 1 and 2.

Fraction	Mn [g/mol]	Mw [g/mol]	Mz [g/mol]	Đ
1: EtOH soluble	1300	2000	2800	1.48
1: EtOH insoluble	4100	13400	2800	3.29
2: EtOH soluble	1600	2400	3500	1.54

2: EtOH insoluble	4300	16000	99200	3.69

5.3 Fractionation cyclic extraction method

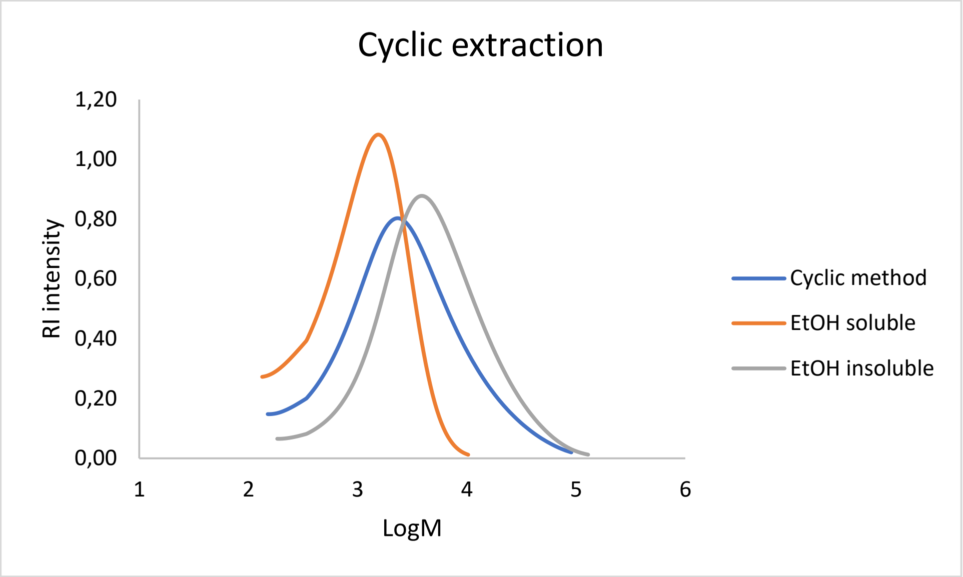

Figure S29. Ethanol fractionation of the cyclic method.

Table S7. Ethanol fractionation of the cyclic method.

**Fraction**	**Mn [g/mol]**	**Mw [g/mol]**	**Mz [g/mol]**	**Đ**
Cyclic method	1200	5600	20900	4.46
EtOH soluble	700	1500	2600	2.15
EtOH insoluble	2300	8400	26000	3.68

5.4 2h and cyclic extraction

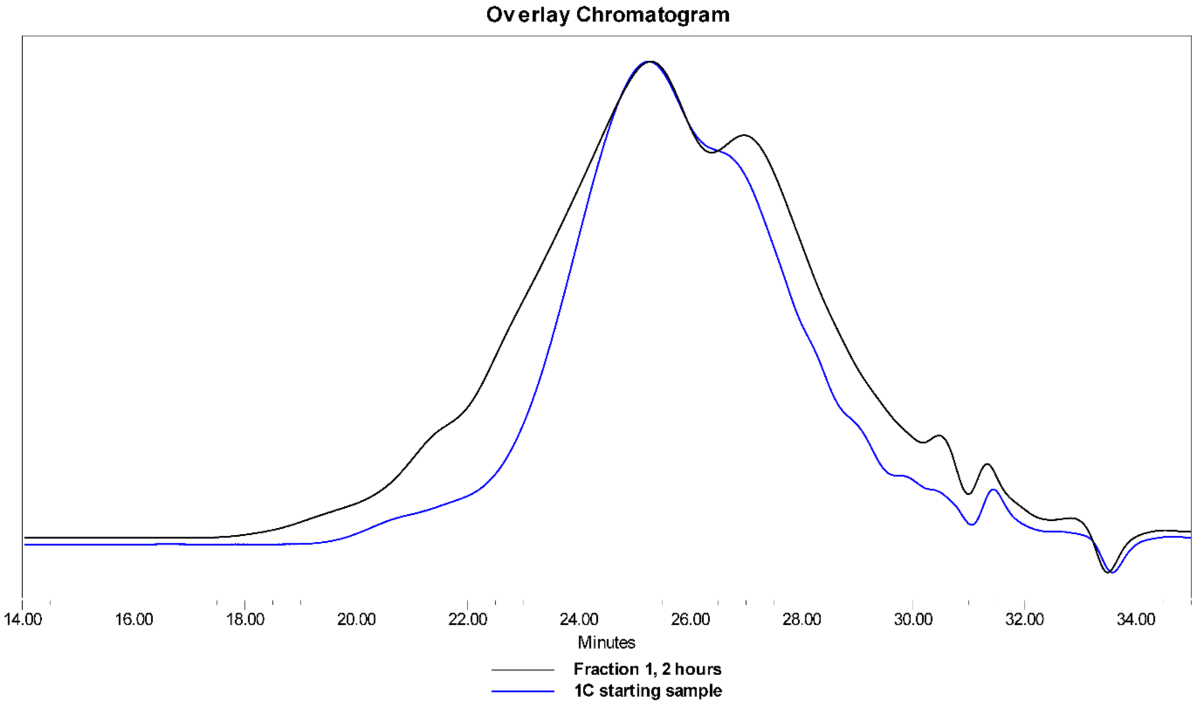

Figure S30. Overlayed chromatogram of 2h and cyclic extracted samples.

5.5 Hemicellulose fraction

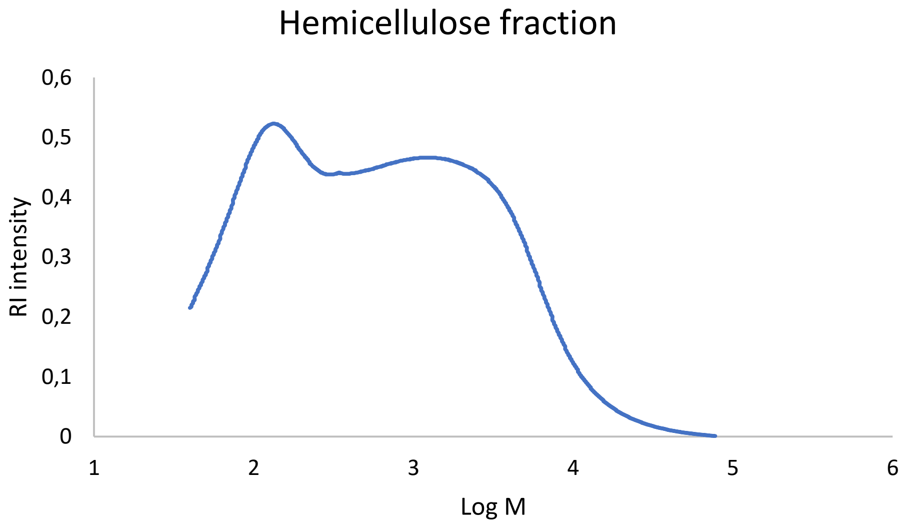

Figure S31. Chromatogram of the hemicellulose fraction.

6 Carbohydrate analysis

The method of the carbohydrate analysis was performed according to the “Carbohydrate analysis” section.

Table S8. Acid hydrolysis. Monosaccharide composition within samples presented as % of the absolute mass (i.e. compared with the start mass of the fraction). Mean ± SD. n=2

Fraction	Ara	Gal	Glu	Xyl	Man	Total
			% of absolute mass			
Spruce wood	1.1 ± 0.0087	1.9 ± 0.013	43.0 ± 0.033	5.8 ± 0.092	11.3 ± 0.20	63.2 ± 0.24
HW extract	4.7 ± 0.26	7.5 ± 0.23	10.1 ± 0.0047	12.5 ± 0.018	34.6 ± 0.086	69.4 ± 0.56
Fiber residue	n.d.	n.d.	71.4 ± 1.1	2.5 ± 0.11	3.0 ± 0.16	76.9 ± 0.81

7 Lignin analysis (KL and ASL)

The method of the lignin analysis was performed according to the “Carbohydrate analysis” section.

Table S9. Lignin analysis. The lignin samples presented as % of the absolute mass (i.e. compared with the start mass of the fraction). Mean ± SD. n=2

Fraction	KL [m%]	ASL [m%]	Total lignin [m%]	Lost [m%]
		% within the fraction		
Spruce Wood	30.8 ± 1.2	0.54 ± 0.0062	31.4 ± 1.2	5.5 ± 0.93
HW extract	8.2 ± 0.092	3.7 ± 0.10	12.0 ± 0.012	18.7 ± 0.57
Fiber residue	22.5 ± 0.98	0.64 ± 0.0058	23.1 ± 0.99	0.016 ± 0.18

8 Collected results

Table S10. Collected results from the different extraction series.

Process	HW extract [m%]	Fiber [m%]	Lignin [m%]	Yield lignin[a] [%]	β-O-4´ level (per 100 Ar)
OS extraction 2h	n.a.	n.a.	15.2	56.3	7
OS extraction 3h	n.a.	39	18.7	69.4	4
1.5% acid 10 fractions	17.7	51.4±0.928	18.1±0.860	66.9	33
0.5% acid 10 fractions	14.9±2.4	54.1±2.7	11.2±1.1	41.4	34±3.1
Cyclic,15 cycles	15.8	n.a.[b]	14.4	53.3	30

[a]: Including extractives. [b]: n.a: not analyzed

9 0.5% acid series samples

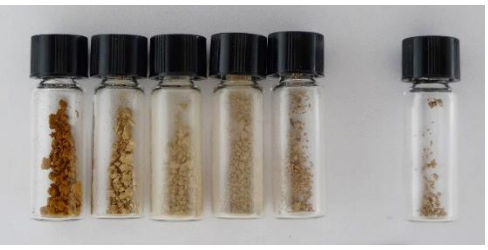

Figure S32. 0.5% acid extraction series of 10 fractions, fraction 1-5 and fraction 10. The images are taken with a white copy paper as a background.
